# Depletion of SNRNP200 inhibits the osteo−/dentinogenic differentiation and cell proliferation potential of stem cells from the apical papilla

**DOI:** 10.1186/s12861-020-00228-y

**Published:** 2020-11-18

**Authors:** Xiaomin Su, Haoqing Yang, Ruitang Shi, Chen Zhang, Huina Liu, Zhipeng Fan, Jianpeng Zhang

**Affiliations:** 1 Laboratory of Molecular Signaling and Stem Cells Therapy, Beijing Key Laboratory of Tooth Regeneration and Function Reconstruction, Capital Medical University School of Stomatology, Beijing, 100050 China; 2 Department of Endodontics, Beijing Stomatological Hospital, School of Stomatology, Capital Medical University, Beijing, 100050 China

**Keywords:** Cell proliferation, Mesenchymal stem cells (MSCs), Osteo−/dentinogenic differentiation, SNRNP200

## Abstract

**Background:**

Tissue regeneration mediated by mesenchymal stem cells (MSCs) is deemed a desirable way to repair teeth and craniomaxillofacial tissue defects. Nevertheless, the molecular mechanisms about cell proliferation and committed differentiation of MSCs remain obscure. Previous researches have proved that lysine demethylase 2A (KDM2A) performed significant function in the regulation of MSC proliferation and differentiation. SNRNP200, as a co-binding factor of KDM2A, its potential effect in regulating MSCs’ function is still unclear. Therefore, stem cells from the apical papilla (SCAPs) were used to investigate the function of SNRNP200 in this research.

**Methods:**

The alkaline phosphatase (ALP) activity assay, Alizarin Red staining, and osteogenesis-related gene expressions were used to examine osteo−/dentinogenic differentiation potential. Carboxyfluorescein diacetate, succinimidyl ester (CFSE) and cell cycle analysis were applied to detect the cell proliferation. Western blot analysis was used to evaluate the expressions of cell cycle-related proteins.

**Results:**

Depletion of SNRNP200 caused an obvious decrease of ALP activity, mineralization formation and the expressions of osteo−/dentinogenic genes including RUNX2, DSPP, DMP1 and BSP. Meanwhile, CFSE and cell cycle assays revealed that knock-down of SNRNP200 inhibited the cell proliferation and blocked cell cycle at the G2/M and S phase in SCAPs. In addition, it was found that depletion of SNRNP200 up-regulated p21 and p53, and down-regulated the CDK1, CyclinB, CyclinE and CDK2.

**Conclusions:**

Depletion of SNRNP200 repressed osteo−/dentinogenic differentiation potentials and restrained cell proliferation through blocking cell cycle progression at the G2/M and S phase, further revealing that SNRNP200 has crucial effects on preserving the proliferation and differentiation potentials of dental tissue-derived MSCs.

**Supplementary Information:**

The online version contains supplementary material available at 10.1186/s12861-020-00228-y.

## Background

Mesenchymal stem cells (MSCs) possess the characteristics of immunoregulation, multi-directional differentiation potential, easy access, rapid proliferation in vitro, low activity loss after cryopreservation, low immunogenicity and non-toxic side effects. Therefore, they have become the most commonly used seed cells for repairing damaged tissue in tissue engineering [1]. Thus far, different sorts of dental tissue-derived MSCs have been separated and identified, including dental pulp stem cells (DPSCs), exfoliated deciduous teeth stem cells (SHEDs), stem cells from the apical papilla (SCAPs), periodontal ligament stem cells (PDLSCs), dental follicle progenitor cells (DFSCs), gingival MSC (GMSCs) and tooth germ progenitor cells (TGPCs) [2, 3]. Simultaneously, researchers have successfully used dental tissue-derived MSCs to regenerate biological roots and periodontal tissues [4]. Nevertheless, the underlying regulatory mechanisms of MSCs self-renewal, proliferation, and directed differentiation are still unknown which limits its clinical application. The formation of certain tissues and the production of a sufficient number of cells depend on the expression of specific genes and the activation of sequential signals. Understanding these signals is conducive to regeneration of desired tissues. So, it is essential to explore the molecular regulation mechanisms of MSCs.

Epigenetic regulation controls MSCs’ fate determination, such as stemness maintenance, differentiation, trans-differentiation and senescence of MSCs [5]. In recent investigations, epigenetic regulation is crucial in the MSCs differentiation and the maintenance of MSCs homeostasis. It has been proved that DNA methylation and histone modifications, the patterns of epigenetics, have significant effects on the MSCs differentiation to specific lineages. Notably, epigenetic dysregulation can lead to aberrations in MSCs function and be associated with human diseases [6, 7]. KDM2A, as a lysine (K)-specific histone demethylase, could selectively remove mono- and di-methylation from histone H3K36 and regulate H3K4me3. Several reports show that KDM2A influence cell proliferation, differentiation, senescence, apoptosis and tumorigenesis by exhibiting their H3K36 demethylase functions at specific genes sites [8, 9]. KDM2A weakened osteo−/dentinogenic differentiation potential of MSCs via the combination with BCOR, then demethylating the histones in Epiregulin promoter to repress EREG transcription [10]. Moreover, the silence of KDM2A can increase the methylation of histones H3K4 and H3K36 on the SFRP2 promoter to upregulate the transcription of SFRP2 [11]. It is found that hypoxia conditions could lead to increasing expression of KDM2A, and suppress SFRP2 transcription by regulating histone methylation in the promoter region of SFRP2 [12]. Evidence has shown that KDM2A negatively regulates cell growth through suppressing ribosomal RNA transcription and drives lung tumorigenesis via epigenetically promoting ERK1/2 signal transduction [13, 14]. In our previous work, we have found that depletion of KDM2A restrained cell proliferation through repressing the expressions of p15^INK4B^ and p27^Kip1^, but improved the adipogenic and chondrogenic differentiation potentials of SCAPs [15, 16]. Nevertheless, the mechanism of KDM2A for regulating the function of MSCs is unclear.

SNRNP200 is a splicing gene of nuclear precursor mRNA. The protein encoded by SNRNP200 is a member of the DEXH-box protein family of RNA helicase, which is widely expressed in the whole body [17]. SNRNP200 is closely connected with the splicing of precursor mRNA, so the defects of SNRNP200 may interfere with the fidelity of transcripts, and then produce abnormal splicing products [18]. And studies have revealed that SNRNP200 participates in antiviral response: SNRNP200 binds virus RNA and interacts with tank binding kinase 1 (TBK1), which promotes the activation of IRF3 and the production of interferon β, thus promoting antiviral response [19]. Meanwhile, SNRNP200 has an important effect on the etiology and pathogenesis of hereditary retinitis pigmentosa, and it has been found that U5 snrnp200 complex is exposed to the leukemic cell membrane in acute myeloid leukemia, which provides a target for tumor treatment [20, 21]. Reports found that SNRNP200 might exert a regulatory effect on cell cycle progression in mammalian cells [22]. Here, we investigated the co-binding proteins of KDM2A in SCAPs with protein mass spectrometry. The results showed that SNRNP200 is a candidate binding partner of KDM2A under hypoxia condition, suggesting that SNRNP200 might regulate the function of MSCs. While, the function of SNRNP200 in MSCs is still unknown.

In our study, we adopted an assay to investigate whether SNRNP200 affects the function of MSCs by using SCAPs, and we found that depletion of SNRNP200 suppressed the osteo−/dentinogenic differentiation and the cell proliferation potentials of SCAPs. Our existing research results revealed that SNRNP200 made pivotal contributions to maintain the proliferation and differentiation potential of dental tissue-derived MSCs.

## Results

### Identification SNRNP200 as the binding protein of KDM2A in SCAPs

To clarify the molecular mechanism by which KDM2A regulates MSCs function, protein mass spectrometry was used to detect proteins in SCAPs that may interact with KDM2A. And the results showed that 19 candidate ligand proteins were differentially expressed in SCAPs in hypoxia group compared to normoxic group (fold change> 1.5) (Supplementary Table 1). Based on the molecular size and peptide compatibility, we selected SNRNP200 for following research.

**Fig. 1:**
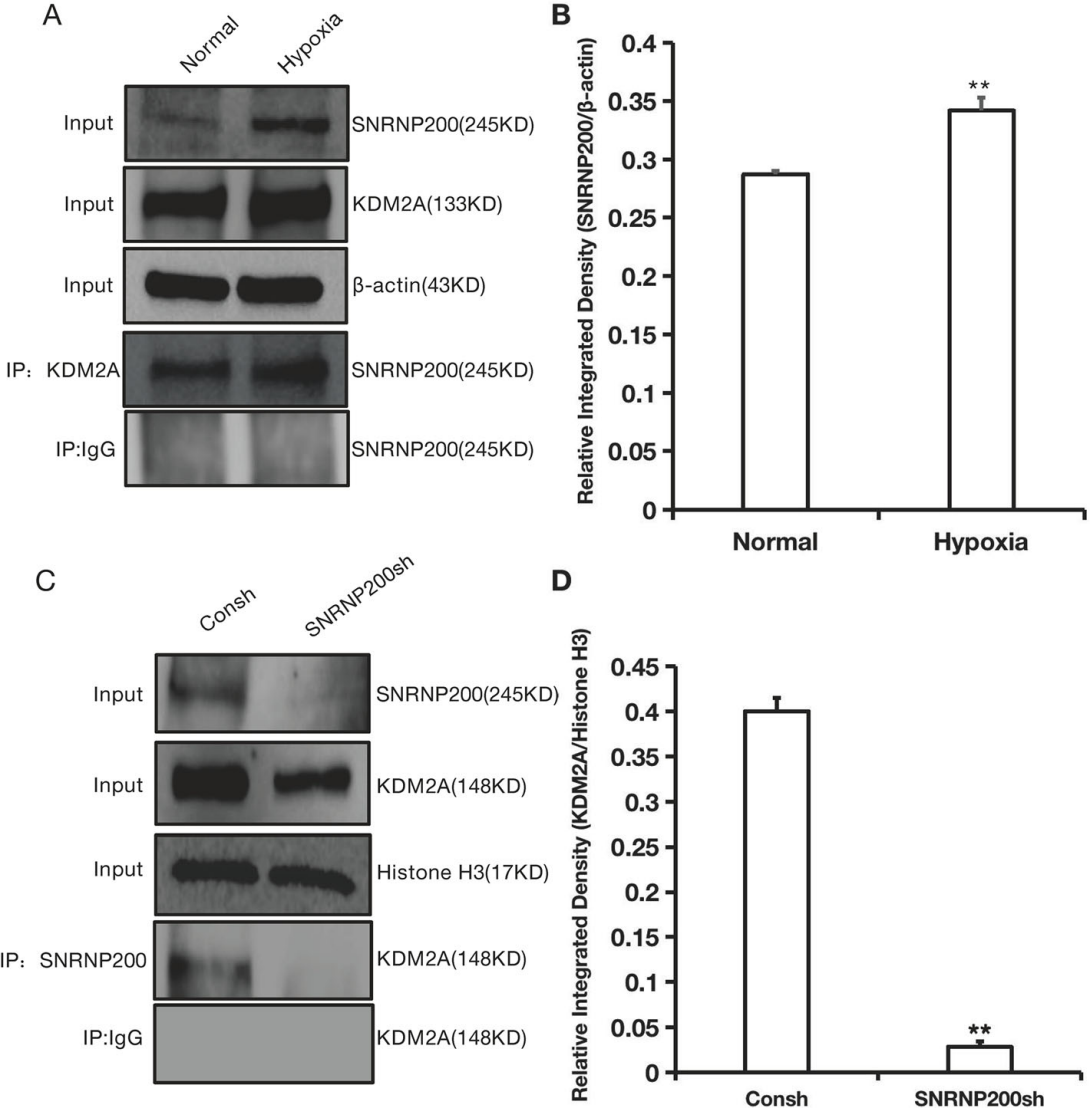
SNRNP200 is a binding partner of KDM2A in SCAPs. **a** Co-IP and Western blot results showed the SNRNP200-KDM2A complexes in SCAPs under hypoxia conditions. **b** Quantitative assessment of SNRNP200 which precipitated by KDM2A antibody between normoxia and hypoxia groups. Input β-actin was used as an internal reference protein. **c** Co-IP and Western blot results showed the SNRNP200-KDM2A complexes in SNRNP200 depleted SCAPs. **d** Quantitative assessment of KDM2A which precipitated by SNRNP200 antibody between control and SNRNP200-depleted groups. Input Histone H3 was used as an internal reference protein

To verify the interactional relations between SNRNP200 and KDM2A, the Co-IP assay was used in SCAPs after cultured under hypoxia. The Western blot and Co-IP analysis suggested that the expression of SNRNP200 was increased and there were more SNRNP200-KDM2A protein complex formations in SCAPs under 3% hypoxia conditions compared with normoxia (Fig. 1a, b; original images of Western blot were in Figures S1, S2, S3 and S4). Then, Co-IP results also revealed that knock-down of SNRNP200 reduced the formation of SNRNP200-KDM2A protein complex in SCAPs (Fig. 1c, d; original images of Western blot were in Figures S5, S6 and S7).

### The knock-down of SNRNP200 inhibited the osteo−/dentinogenic differentiation potential in SCAPs

**Fig. 2:**
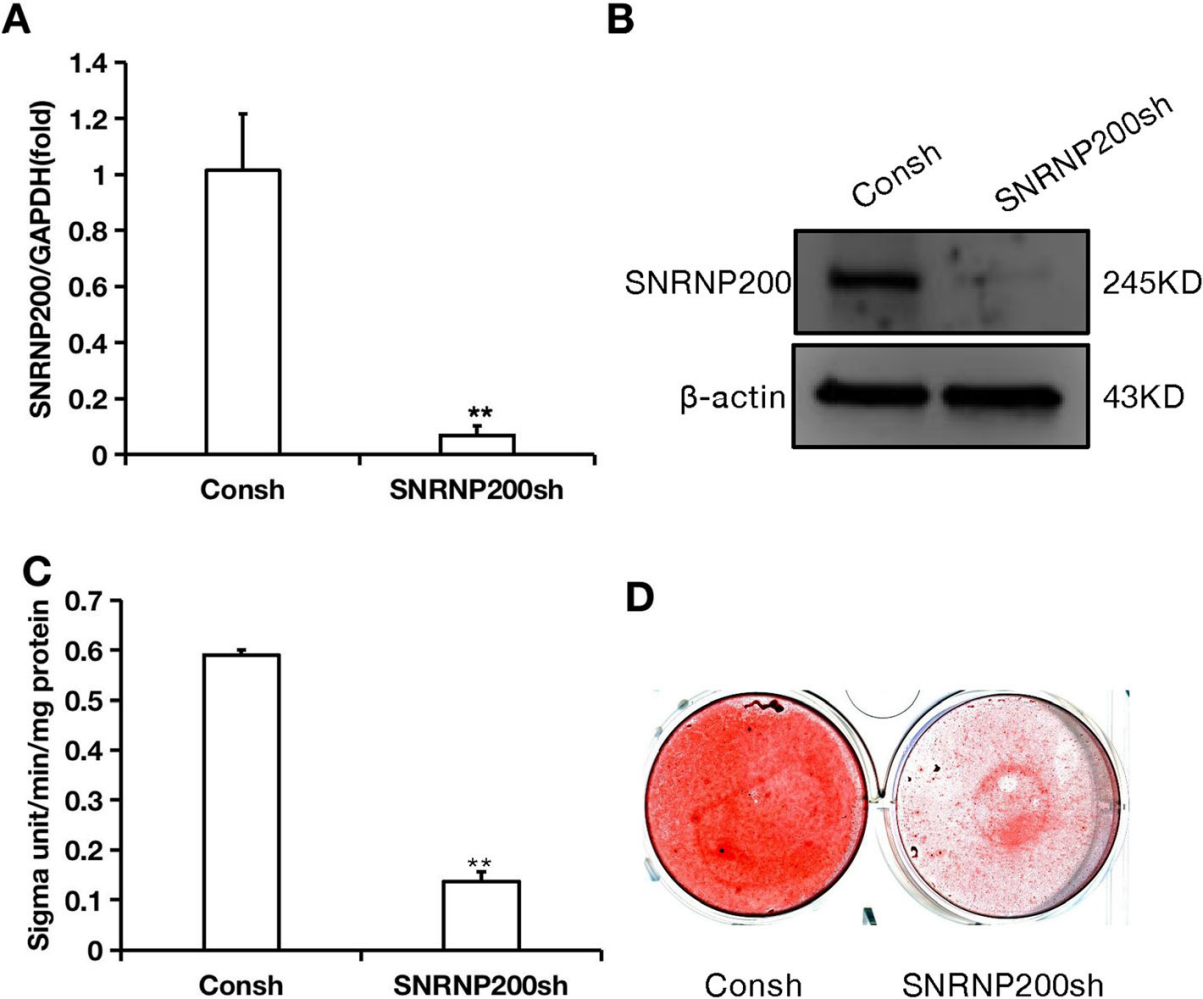
Depletion of SNRNP200 inhibited the potential of osteo-/dentinogenic differentiation in SCAPs. **a** Real-time RT-PCR showed the depletion of SNRNP200 expression. GAPDH was used as an internal control. **b** Western blot results showed that SNRNP200 was knocked-down in SCAPs. β-actin was used as an internal control. **c** SNRNP200 depletion reduced the ALP activity in SCAPs. **d** Alizarin red staining showed that SNRNP200 depletion reduced mineralization in SCAPs. Student’s t-test was used to analyze the statistical significance. All error bars represent SD (*n* = 3). **P ≤ 0.01

**Fig. 3:**
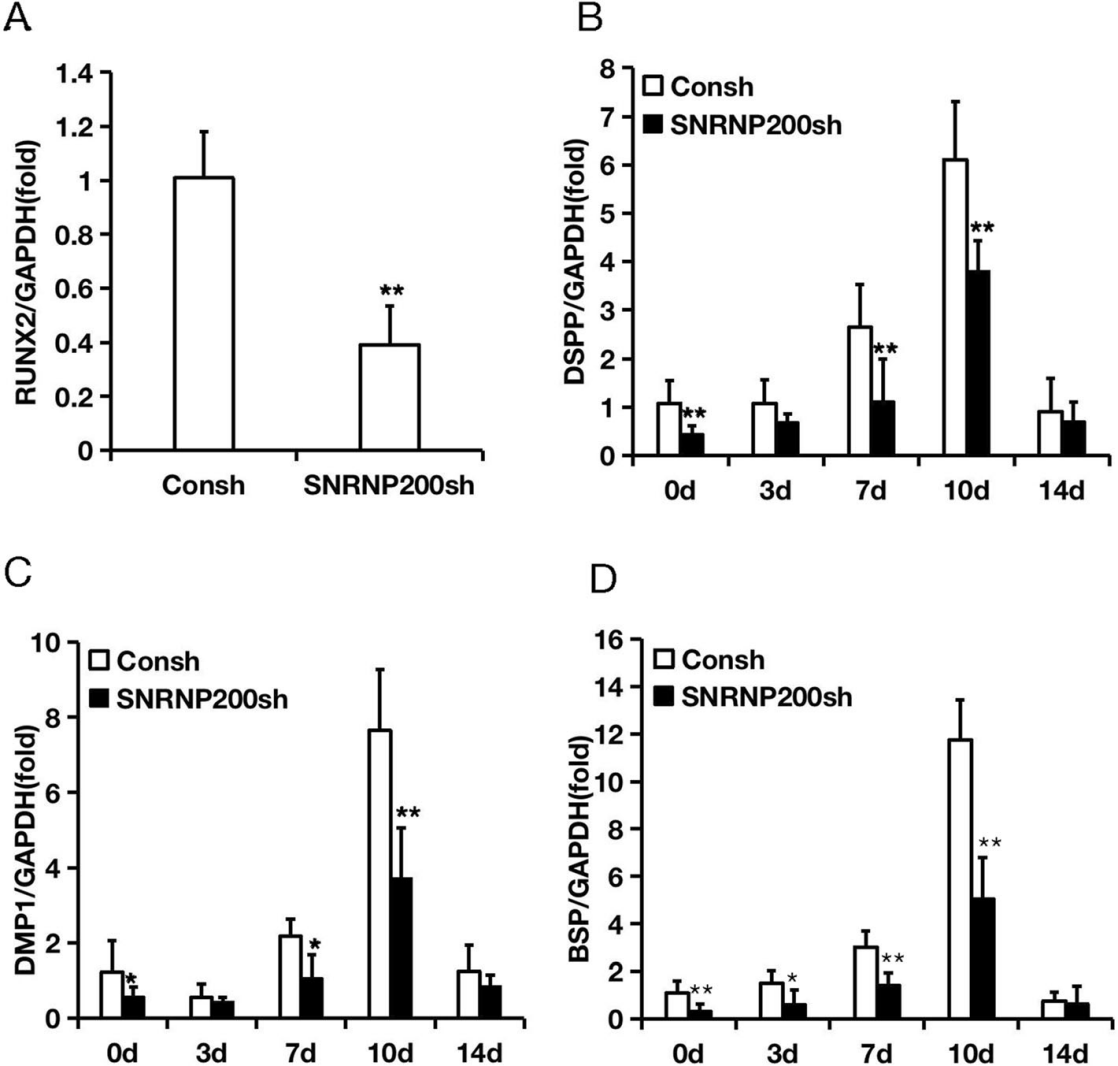
Depletion of SNRNP200 reduced the expression of osteo−/dentinogenic differentiation genes in SCAPs. **a**-**d** Real-time RT-PCR showed that SNRNP200 depletion causes a decrease in the expression of RUNX2 (**a**), DSPP (**b**), DMP1 (**c**) and BSP (**d**). GAPDH was used as an internal reference gene. Student’s t-test was used to analyze statistical significance. All error bars represent SD (*n* = 3). *P ≤ 0.05. **P ≤ 0.01

To identify the role of SNRNP200 in SCAPs, the shRNA targeting to SNRNP200 was transfected into SCAPs through lentiviral infection. Then we tested the knock-down efficiency by the real-time RT-PCR (Fig. 2a) and Western blot (Fig. 2b; original images of Western blot were in Figures S8 and S9). Subsequently, we detected whether SNRNP200 could affect the osteo−/dentinogenic differentiation of SCAPs. After 3 days of osteo−/dentinogenic inducing, we found that the ALP activity was obviously decreased in SNRNP200-depleted cells. (Fig. 2c). After induction for 2 weeks, the Alizarin Red staining indicated the mineralization was weakened in SNRNP200-depleted SCAPs compared with the matched control (Fig. 2d). Furthermore, before and during the mineralization induction, real-time RT-PCR was used to analyze the expression level of osteo−/dentinogenic genes including RUNX2, DSPP, DMP1 and BSP. The results displayed that the expression of RUNX2, DSPP, DMP1 and BSP in the SNRNP200-depleted SCAPs was down-regulated compared to that in the matched control before mineralization induction (0 day). The expressions of DSPP and DMP1 were decreased at 7 and 10 days, the BSP expression was down-regulated at 3, 7 and 10 days after induction in SNRNP200-depleted SCAPs compared with the matched control (Fig. 3).

### The knock-down of SNRNP200 inhibited the cell proliferation potential and promoted cell senescence in SCAPs

**Fig. 4:**
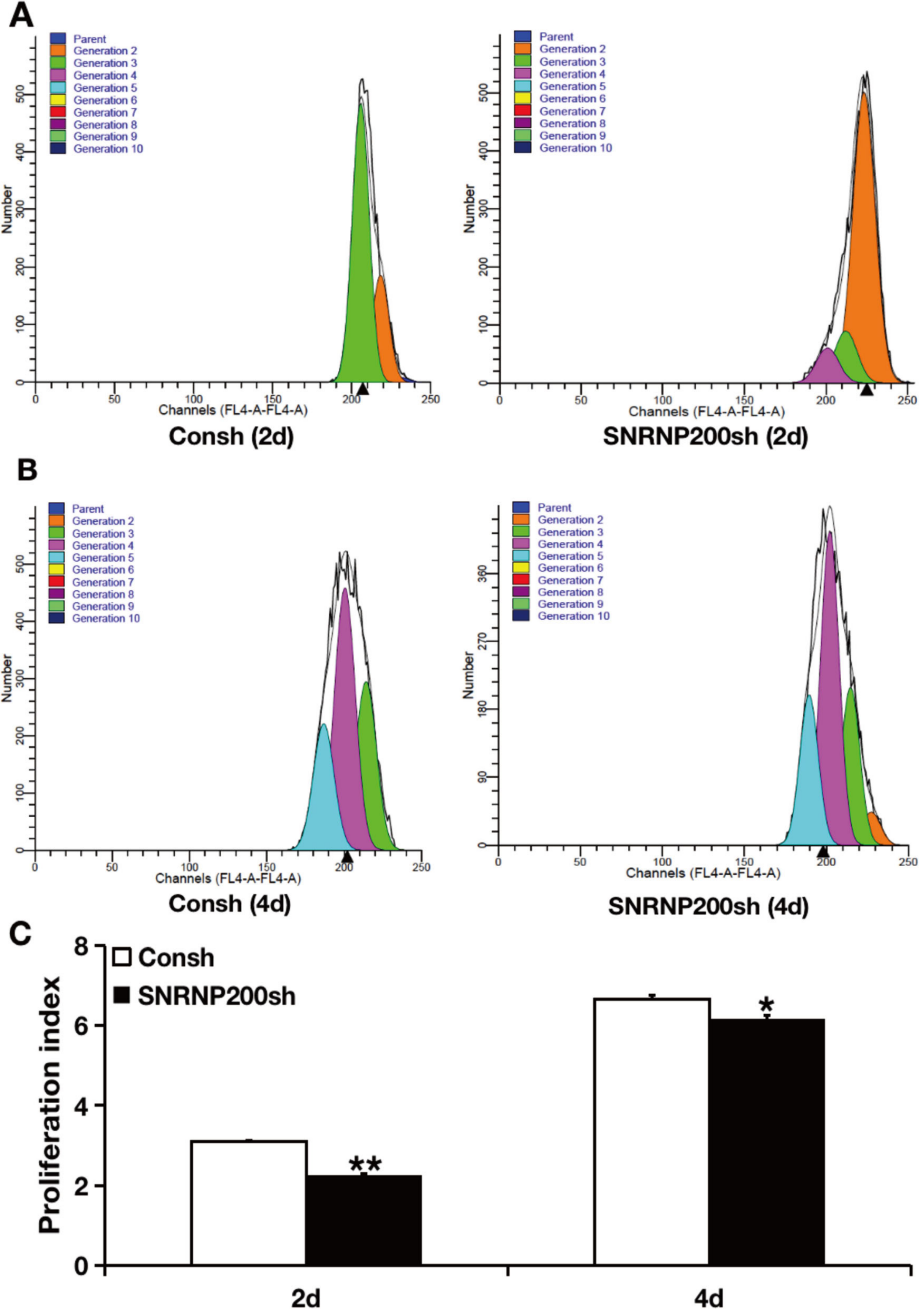
Depletion of SNRNP200 suppressed the cell proliferation of SCAPs. **a** The CFSE assay results at 2 day. **b** The CFSE assay results at 4 days. **c** The quantitative analysis results of CFSE assay. Student’s t- test was used to analyze the statistical significance. All error bars represent SD (*n* = 3). *P ≤ 0.05. **P ≤ 0.01

**Fig. 5:**
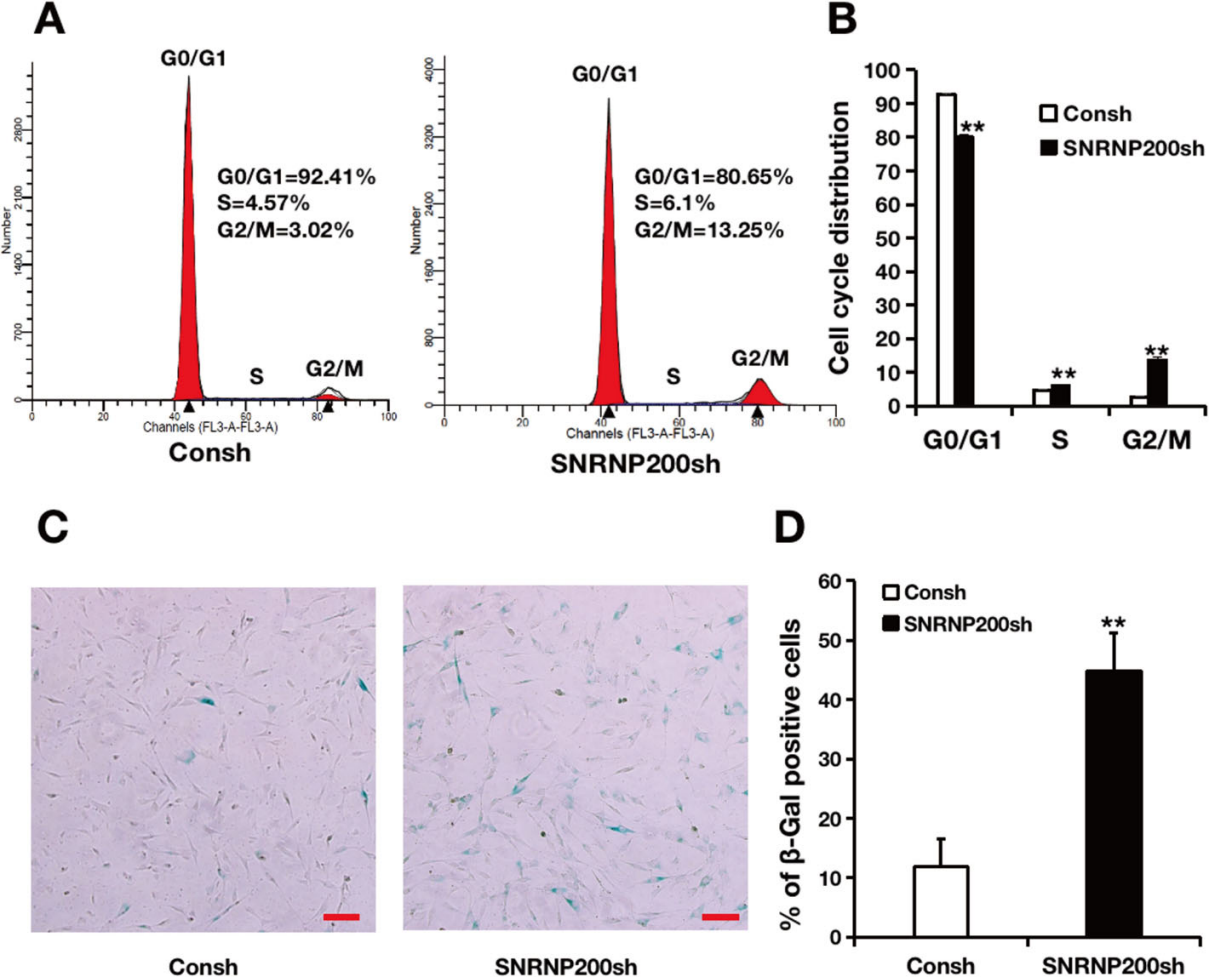
Depletion of SNRNP200 affected the cell cycle and cell senescence in SCAPs. **a**, **b** The flow cytometry assay (**a**) and quantitative analysis (**b**) revealed that SNRNP200 depletion arrested cell cycle at G2/M and S phase. **c**, **d** The β-Gal staining (**c**) and quantitative analysis (**d**) results. Student’s t-test was used to analyze the statistical significance. All error bars represent SD (*n* = 3). **P ≤ 0.01. Scale bar = 100 μm

To explore whether SNRNP200 affected the cell proliferation, we obtained stably transfected cells after 3-day puromycin selection and labeled them with CFSE. Then the labeled cells were cultured for 2 and 4 days to detect its proliferation. The CFSE assay and quantitative analysis results displayed that knock-down of SNRNP200 leads to a decrease in the capacity of cell proliferation compared with the matched control (Fig. 4). To further confirm the effect of SNRNP200 on cell cycle distribution in SCAPs, the cell cycle analysis was carried out. The results of flow cytometry detection and quantitative analysis showed that the cell percentage in G0/G1 phase decreased, while cell percentage in G2/M and S phase increased in SNRNP200-depleted SCAPs compared with the matched control (Fig. 5a, b). In addition, the β-Gal staining and quantitative analysis results explored that SA-β-gal positive cell numbers were increased in SNRNP200 depleted SCAPs compared to the matched control (Fig. 5c, d).

### The knock-down of SNRNP200 promoted the expressions of p21 and p53 and inhibited the expressions of Cyclin B, CDK1, Cyclin E and CDK2 in SCAPs

**Fig. 6:**
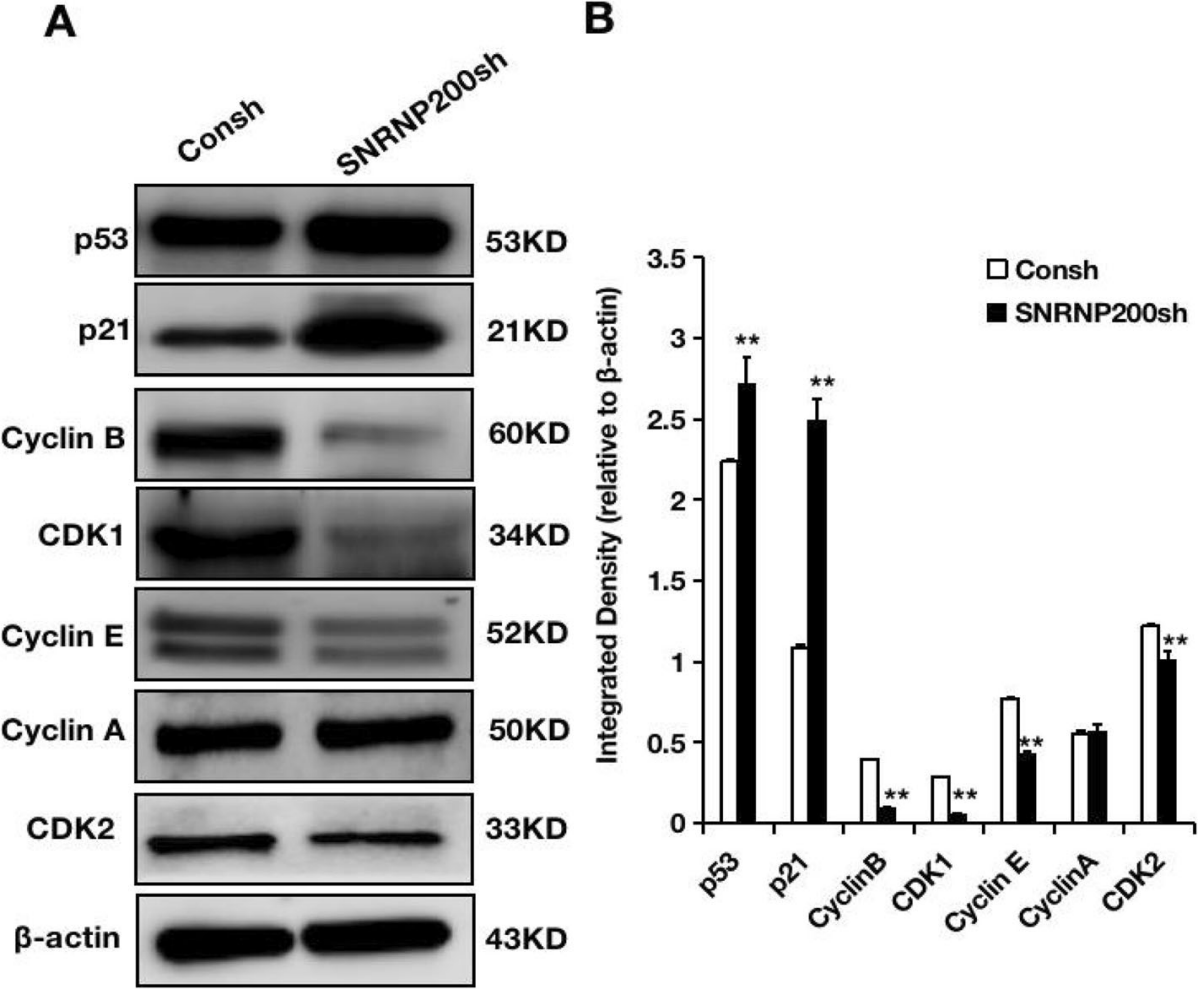
Depletion of SNRNP200 regulated the proteins related to the cell cycle and cell senescence in SCAPs. **a** The Western blot results showed that the expressions of p53, p21, CyclinB, CDK1, Cyclin E, Cyclin A and CDK2 in SCAPs. **b** Quantitative analysis of Western blot results. β-actin was used as an internal reference protein. Student’s t-test was used to analyze the statistical significance. All error bars represent SD (*n* = 3). *P ≤ 0.05. **P ≤ 0.01

To elucidate how SNRNP200 acts on the cell cycle and cell senescence of SCAPs, we analyzed the expression of p21, p53, CyclinB, CDK1, CyclinE, CDK2 and CyclinA. Western blot analysis suggested that the expression of p21 and p53 was obviously increased in SNRNP200-depleted SCAPs, and the expression of CyclinB, CDK1, CyclinE, CDK2 were appreciably decreased in SNRNP200-depleted SCAPs, and the expression of CyclinA was not significantly changed in SNRNP200-depleted SCAPs compared with the matched control (Fig. 6; original images of Western blot were in Figures S10, S11, S12, S13, S14, S15 and S16).

## Discussion

As a kind of dental tissue-derived MSCs, the SCAPs are used for tissue regeneration due to their specific advantages. For example, SCAPs have stable biological and immunological characteristics, higher proliferation rate, longer telomere length, higher expression of survivin, stronger telomerase activity and excellent multi-directional differentiation potential [23]. However, some issues need to be addressed before application, especially concerning the limited tissue origin, the unclear molecular mechanisms about directed differentiation and proliferation of SCAPs. Our previous studies have suggested KDM2A could be important for regulating the proliferation and osteo−/dentinogenic differentiation of SCAPs. To further expose the underlying molecular mechanisms, we discovered several potential candidate partners for interaction with KDM2A by mass spectrometry, such as MYH9, LRP1 and SNRNP200. MYH9, a cytoplasmic myosin expressed in most cells and tissues, plays a major role in human development and disease [24]. LRP1 is a cell surface receptor that is widely expressed in many cells and can regulate tumor cell migration and cellular cholesterol homeostasis [25, 26]. SNRNP200, a splicing factor, showed a 3-fold increase in expression on the cell surface when MSCs were induced to differentiate towards osteoblasts [27]. In our study, we confirmed that SNRNP200 is an interacting protein of KDM2A and further investigated the effect of SNRNP200 on the function of MSCs.

In this paper, we discovered that knock-down of SNRNP200 weakened the ALP activity, the mineralization formation and the expressions of RUNX2, BSP, DSPP and DMP1. During the progression of osteo−/dentinogenic differentiation, various osteo-/dentinogenic markers reflect the strength of osteo-/dentinogenic ability in MSCs. RUNX2 is the early osteogenic associated gene. Several studies have shown that the RUNX2 is a major regulatory factor for the osteo−/dentinogenic differentiation of MSCs which guides MSCs to differentiate into pre-osteoblast [28–30]. Meanwhile, investigation discovered that the higher the ALP activity, a marker of early osteogenesis, the stronger ability of pre-osteoblasts differentiating to mature osteoblasts [31, 32]. Bone sialoprotein (BSP), dentin sialophosphoprotein (DSPP) and dentin matrix protein 1 (DMP1) are the members of the Small Integrin Binding Ligand N-linked Glycoprotein (SIBLING) family [33]. BSP belongs to extracellular matrix (ECM) proteins of mineralized tissues, which is closely associated with cell differentiation and mineralization [34]. DSPP is a precursor protein that is expressed in dentin matrix and implicated in dentin formation [35]. DMP1 is a non-collagenous bone matrix protein produced exclusively by osteocytes and is related to the mineralization of bones and teeth [36]. In addition, BSP, DSPP and DMP1 are genetic markers at middle and late stage of osteo−/dentinogenic differentiation [37]. These results suggested that inhibition of SNRNP200 will impair the osteo−/dentinogenic differentiation potentials of SCAPs might via the key transcript factor, RUNX2.

In our study, it was also found that depletion of SNRNP200 restrains cell proliferation and blocks cell cycle at the G2/M and S phase of SCAPs. Under the precise and strict regulation of various regulatory factors on cell cycle, the cell cycle process can be realized. The core of these regulatory factors is cyclin-dependent kinase (CDK), cyclin-dependent kinase inhibitors (CDKIs), a negative regulator of CDK, and the cyclin protein, a positive regulator of CDK [38]. CDK4-CyclinD complexes and CDK6-CyclinD complexes are involved in the G1 progression, and CDK2-CyclinE complexes and CDK3-CyclinA complexes are considered to act on the transition from G1 to S phase [39]. During S phase, CyclinA-CDK2 complex is necessary for mammalian DNA replication [40, 41]. And CyclinE/CDK2 activity seems to be related to triggering initiation of DNA replication [42]. The activation of CDK1-CyclinB complexes is necessary for the initiation of mitosis and cell cycle was put into G2/M phase [43]. In addition, several CKIs that regulate cell cycle can be classified into two types. One type of CKIs is INK4 family member including p16^INK4a^, p15^INK4b^, p18^INK4c^ and p19^INK4d^ which mostly focus on CDK4 and CDK6. Other is Cip/Kip family member including p21^WAF1/Cip1^, p27^Kip1^, and p57^Kip2^ which restrain the activity of CyclinA-, CyclinD-, CyclinB- and CyclinE-dependent kinase complexes [44]. Researches demonstrated p21^WAF1/Cip1^, the transcriptional targets of p53, can inhibit CDK1 directly leading to G2 arrest [45, 46]. At the level of cell cycle, the pathways of p53/p21^WAF1/Cip1^ and p16^Ink4a^/retinoblastoma protein have essential functions on irreversible growth arrest and cellular senescence [47]. In our study, SNRNP200 depletion suppressed the expression of CyclinB, CDK1, CyclinE, and CDK2, indicated that SNRNP200 depletion blocked cell cycle at the S phase may owning to the inhibition of CDK1, CDK2, and CyclinE. And decreased expression of CyclinB and CDK1 would reduce the activity CDK1-CyclinB complexes, contributes to G2/M phase arrest after depletion of SNRNP200. Moreover, SNRNP200 depletion up-regulated the expression of p53 and p21^WAF1/Cip1^, indicated that impaired CDK1-CyclinB complex might through enhanced expression of p53 and p21^WAF1/Cip1^ during G2/M phase arrest.

Moreover, recent reports discovered that MSCs exhibited the diminished proliferation and differentiation ability with senescence [48, 49]. Senescent cells finally exhausted their ability to divide, performed hypertrophic morphology, showed positive staining for SA-β-Gal, increased generation of ROS, and increased the production of lipofuscin [50]. And the higher levels of p53 and p21^WAF1/Cip1^ result in the cell senescence. Our study also suggested that depletion of SNRNP200 induced the SA-β-Gal positive staining in SCAPs and enhanced the expression of p53 and p21^WAF1/Cip1^, indicated depletion of SNRNP200 enhanced the senescence of SCAPs. Previous research proposed that proliferating cells can be induced to senescence by the increasing of cell-cycle arrest signals, while damaged quiescent or differentiated cells could be senescent under the stimulation of expansion [48]. In summary, our investigation suggested that the depletion of SNRNP200 contributed to vital cell cycle arrest and cell senescence, and weakened the capabilities of proliferation and differentiation potentials in SCAPs.

Oxygen is a vital factor in the stem cell niche in vivo. MSCs-mediated tissue repair and regeneration are usually carried out under the condition of hypoxia. And hypoxia affect the proliferation and osteogenic differentiation potentials of MSCs compared with normoxia [51, 52]. Our previous research suggested that hypoxia conditions promote the expression of KDM2A [12]. And depletion of KDM2A also inhibit the cell proliferation [15]. Our results showed that KDM2A and SNRNP200 could form protein complexes and more SNRNP200-KDM2A complexes formed under hypoxia. These indicated that SNRNP200 might regulate the cell proliferation by associated with KDM2A, and their function might be enhanced under the hypoxia. But KDM2A impaired the osteo−/odontogenic differentiation potential of SCAPs, which would have an unfavorable effect on tissue regeneration [12]. And our present studies revealed that depletion of SNRNP200 weakened the osteo−/odontogenic differentiation potential of SCAPs. These indicated the functions of SNRNP200 on osteo−/odontogenic differentiation would depend on other partners, further investigation will be performed to elucidate this point.

## Conclusions

To sum up, our findings suggested that depletion of SNRNP200 weakened the cell proliferation abilities and osteo−/dentinogenic differentiation potentials of SCAPs. What’s more, inhibition of SNRNP200 impaired the osteo−/dentinogenic differentiation potentials of SCAPs might via key transcript factor, RUNX2. And SNRNP200 depletion inhibits cell proliferation via blocking cell cycle in G2 /M and S phases, and accelerating the aging process enhanced these effects. Through our study, the role of SNRNP200 in preserving the proliferation and differentiation potentials of dental tissue-derived MSCs was put forward, which lays a foundation for further exploring the regulatory mechanisms about MSCs’ directed differentiation and offers the potential target for promoting tissue repair and regeneration.

## Methods

### Protein mass spectrometry

The extracted proteins were separated by gel electrophoresis. Then the gel was stained and cut for protein mass spectrometry analysis. The detailed procedures refer to our previous research [53].

### Cell culture

All the experiments based on human stem cells were informed by the ISSCR “Guidelines for the Conduct of Human Embryonic Stem Cell Research”. The healthy teeth tissues were acquired from patients with informed consent and under approved guidelines set by the Beijing Stomatological Hospital, Capital Medical University. The isolation, culture, and identification of primary cells are as described in our previous studies [11]. The cultured SCAPs at passage 3–5 were applied in following research.

### Plasmid construction and viral infection

The SNRNP200-specific short hairpin RNAs (SNRNP200 shRNA) and Control shRNA were imported into the LV2 lentiviral vectors (Genepharma company, Suzhou, China). When growing to a suitable density, the SCAPs were transfected with lentiviruses in culture medium with polybrene (6 μg/mL, Sigma-Aldrich, St. Louis, MO, USA) for 12 h. After 48 h, the SCAPs transfected with viruses were screened with 2 μg/mL puromycin for 3 days. The shRNA sequences were SNRNP200 shRNA (SNRNP200sh), 5′GCCTACCTCTATATCCGAATG-3′, and Control shRNA (Consh), 5′-TTCTCCGAACGTGTCACGTTTC-3′.

### Reverse transcriptase-polymerase chain reaction (RT-PCR) and real-time RT-PCR

The whole RNA in SCAPs was abstracted by Trizol reagents (Invitrogen). Then we use part of total RNA to synthesize cDNA and the same amounts of cDNA and primers were applied for the real-time amplification of target genes. The details of procedures referred to our previous study [10]. The primer sequences used were listed in Supplementary Table 2.

### Western blot analysis

In the presence of RIPA lysis buffer, cells are lysed and total proteins are extracted. Basic experiment procedures of Western blot were consistent with our previous methods [11]. The introductions of primary antibodies were as follows: SNRNP200 (Cat No. ab176715, Abcam), CyclinA (Cat No. SAB4503499, Sigma-Aldrich), CDK2 (Cat No. 05–363, Merck Millipore), CyclinD (Cat No. 05–137, Merck Millipore), CDK1 (Cat No. 19532-I-AP, Protech), p21^WAF1/Cip1^ (cyclin-dependent kinase inhibitor 1A), (Cat No. 10355-I-AP, Protech), p53 (Cat No.10442-I-AP, Protech), CyclinB (Cat No.55004-I-AP, Protech) and CyclinE (Cat No. 05–363, Merck Millipore, Darmstadt, Germany). The housekeeping protein we used was Histone H3 (Cat No. Sc-10,809, SantaCruz) or beta-actin (β-actin; Cat. No. ab129348, Abcam, Cambridge, UK). Image J was applied to the quantitative analysis of proteins.

### Alkaline phosphatase activity assay and alizarin red staining

SCAPs were induced in mineralization-inducing medium for 3 days, ALP activity was tested as shown in our past study [11]. After 2 weeks of osteogenic inducing, the mineralized cells were fixed with 70% ethanol and then stained with 2% Alizarin Red (Sigma-Aldrich).

### CFSE assay

SCAPs were labeled by a fluorescent probe referring to the CellTrace™ CFSE Cell Proliferation Kit Protocol (Invitrogen) and then seeded in 6 cm dishes at a density of 2.5 × 10^5^ cells/dish. After 2, 4 days’ culturing, we used 0.25% trypsin to get cells suspension. Then the samples were analyzed by flow cytometry (Calibur; BD Biosciences). Cell proliferation index was assessed by ModFit LT software.

### Cell cycle assay

SCAPs were collected, washed, fixed, stained and finally detected by flow cytometry. The information about fluorescence intensities was analyzed by ModFit LT software. More details about experimental procedures were described as our previous work [15].

### SA-β-gal staining

SCAPs were seeded in 24-well plates at a density of 4 × 10^4^ cells/well. After 12 h culture, cells were stained with GENMED working solution following Cell Senescence Testing Kit instructions (GenMed Scientifics). Cells were incubated at 37 °C for 12 h and then observed under an optical microscope. Image-Pro Plus software was used to count the stained cells.

### Co-immunoprecipitation (co-IP) assay

SCAPs were cultured under hypoxia conditions or infected with lentivirus. Then the cells were washed twice and lysed in cold IP Lysis Buffer (25 mM Tris-HCl pH 7.4, 150 mM NaCl, 1 mM EDTA, 1% NP-40, 5% glycerol and a complete protease inhibitor cocktail) for 20 min on ice. Lysates were centrifuged for 15 min at 4 °C to collect the supernatants. The obtained protein samples were incubated with anti-SNRNP200 antibody (Cat No. ab176715, Abcam), anti-KDM2A antibody (Cat No. ab31739, ab240747 Abcam) or normal rabbit IgG (Cat No. sc-2027, Santa Cruz Biotechnology) at 4 °C overnight. Next day, the protein mixtures addition with A/G magnetic bead (Cat No. HY-K202, MCE) were incubated for 4 h at 4 °C. Finally, the immune compounds were harvested, washed, eluted and detected by Western blot.

### Statistical analysis

All statistical calculations were performed using SPSS10 statistical software. Statistical significance was determined using Student’s t test, with *p* ≤ 0.05 indicating statistical significance.

## Supplementary Information

**Additional file 1:** **Table S1.** The differentially expressed protein between normal group and hypoxia group.

**Additional file 2:** **Table S2.** Primers sequences used in the real-time RT-PCR.

**Additional file 3:** **Figure S1-S4.** Original, full-length gel and blot images of Fig. 1a. **Figure S5-S7.** Original, full-length gel and blot images of Fig. 1c. **Figure S8-S9.** Original, full-length gel and blot images of Fig. 2b. **Figure S10-S16.** Original, full-length gel and blot images of Fig. 6a.

## Data Availability

The dataset used and/or analyzed during the current study available from the corresponding author on reasonable request.

## Abbreviations

SNRNP200: Small nuclear ribonucleoprotein U5 subunit 200
KDM2A: Lysine demethylase 2A
MSCs: Mesenchymal stem cells
SCAPs: Stem cells from the apical papilla
shRNAs: Short-hairpin RNAs
RUNX2: RUNX family transcription factor 2
DSPP: Dentin sialophosphoprotein
DMP1: Dentin matrix acidic phosphoprotein 1
BSP: Bone sialoprotein
