# Supplementary material for: Depletion of SNRNP200 inhibits the osteo−/dentinogenic differentiation and cell proliferation potential of stem cells from the apical papilla
Source: BMC Dev Biol. 2020 Nov 18;20:22. doi: 10.1186/s12861-020-00228-y (PMC7672972; doi:10.1186/s12861-020-00228-y)
Supplement: Supplementary file 1 — Additional file 1: Table S1. The differentially expressed protein between normal group and hypoxia group. [file 12861_2020_228_MOESM1_ESM.docx]

**Supplementary Table 1. The differentially expressed protein between normal group and hypoxia group**

| Accession | Peptide Numbers in Normal Gruop | Peptide Numbers in Hypoxia Gruop | fold change |
| --- | --- | --- | --- |
| sp\|P35579\|MYH9_HUMAN | 7 | 37 | 5.29 |
| sp\|Q07954\|LRP1_HUMAN | 7 | 24 | 3.43 |
| sp\|P11047\|LAMC1_HUMAN | 6 | 13 | 2.17 |
| sp\|P40926\|MDHM_HUMAN | 13 | 6 | 2.17 |
| sp\|P05141\|ADT2_HUMAN | 10 | 5 | 2.00 |
| sp\|Q14204\|DYHC1_HUMAN | 17 | 9 | 1.89 |
| sp\|O75643\|SNRNP200_HUMAN | 6 | 11 | 1.83 |
| sp\|Q9UQ80\|PA2G4_HUMAN | 11 | 6 | 1.83 |
| sp\|Q6P2Q9\|PRP8_HUMAN | 12 | 7 | 1.71 |
| tr\|J3KPS3\|J3KPS3_HUMAN | 10 | 6 | 1.67 |
| tr\|A0A0A0MRV0\|A0A0A0MRV0_HUMAN | 15 | 24 | 1.60 |
| sp\|P07737\|PROF1_HUMAN | 5 | 8 | 1.60 |
| sp\|P11216\|PYGB_HUMAN | 8 | 5 | 1.60 |
| tr\|E9PQN2\|E9PQN2_HUMAN | 5 | 8 | 1.60 |
| sp\|P49257\|LMAN1_HUMAN | 5 | 8 | 1.60 |
| sp\|O00571\|DDX3X_HUMAN | 8 | 5 | 1.60 |
| sp\|O43143\|DHX15_HUMAN | 12 | 19 | 1.58 |
| sp\|Q9NZM1\|MYOF_HUMAN | 19 | 30 | 1.58 |
| sp\|Q8NC51\|PAIRB_HUMAN | 11 | 7 | 1.57 |
