# Supplementary material for: Depletion of SNRNP200 inhibits the osteo−/dentinogenic differentiation and cell proliferation potential of stem cells from the apical papilla
Source: BMC Dev Biol. 2020 Nov 18;20:22. doi: 10.1186/s12861-020-00228-y (PMC7672972; doi:10.1186/s12861-020-00228-y)
Supplement: Supplementary file 2 — Additional file 2: Table S2. Primers sequences used in the real-time RT-PCR. [file 12861_2020_228_MOESM2_ESM.docx]

**Supplementary Table 2. Primers sequences used in the real-time RT-PCR**

| Gene symbol | Primer sequence (5′‐3′) |
| --- | --- |
| GAPDH‐F | CGGACCAATACGACCAAATCCG |
| GAPDH‐R | AGCCACATCGCTCAGACACC |
| SNRNP200‐F | CCAAGCTGACCGTTCTCTCAT |
| SNRNP200‐R | GCCTTGTCTCCCATACGGG |
| RUNX2‐F | TCTTAGAACAAATTCTGCCCTTT |
| RUNX2‐R | TGCTTTGGTCTTGAAATCACA |
| DSPP‐F | CGACATAGGTCACAATGAGGATGTCG |
| DSPP‐R | TTGCTTCCAGCTACTTGAGGTC |
| DMP1‐F | CGTGGACAAAGAAGATAGCAACTCCACG |
| DMP1‐R | TTCCGGCTCTCTATCTCAATGTTT |
| BSP-F | CAGGCCACGATATTATCTTTACA |
| BSP-R | CTCCTCTTCTTCCTCCTCCTC |
