# Supplementary material for: Depletion of SNRNP200 inhibits the osteo−/dentinogenic differentiation and cell proliferation potential of stem cells from the apical papilla
Source: BMC Dev Biol. 2020 Nov 18;20:22. doi: 10.1186/s12861-020-00228-y (PMC7672972; doi:10.1186/s12861-020-00228-y)
Supplement: Supplementary file 3 — Additional file 3: Figure S1-S4. Original, full-length gel and blot images of Fig. 1a. Figure S5-S7. Original, full-length gel and blot images of Fig. 1c. Figure S8-S9. Original, full-length gel and blot images of Fig. 2b. Figure S10-S16. Original, full-length gel and blot images of Fig. 6a. [file 12861_2020_228_MOESM3_ESM.pptx]

## Slide 1
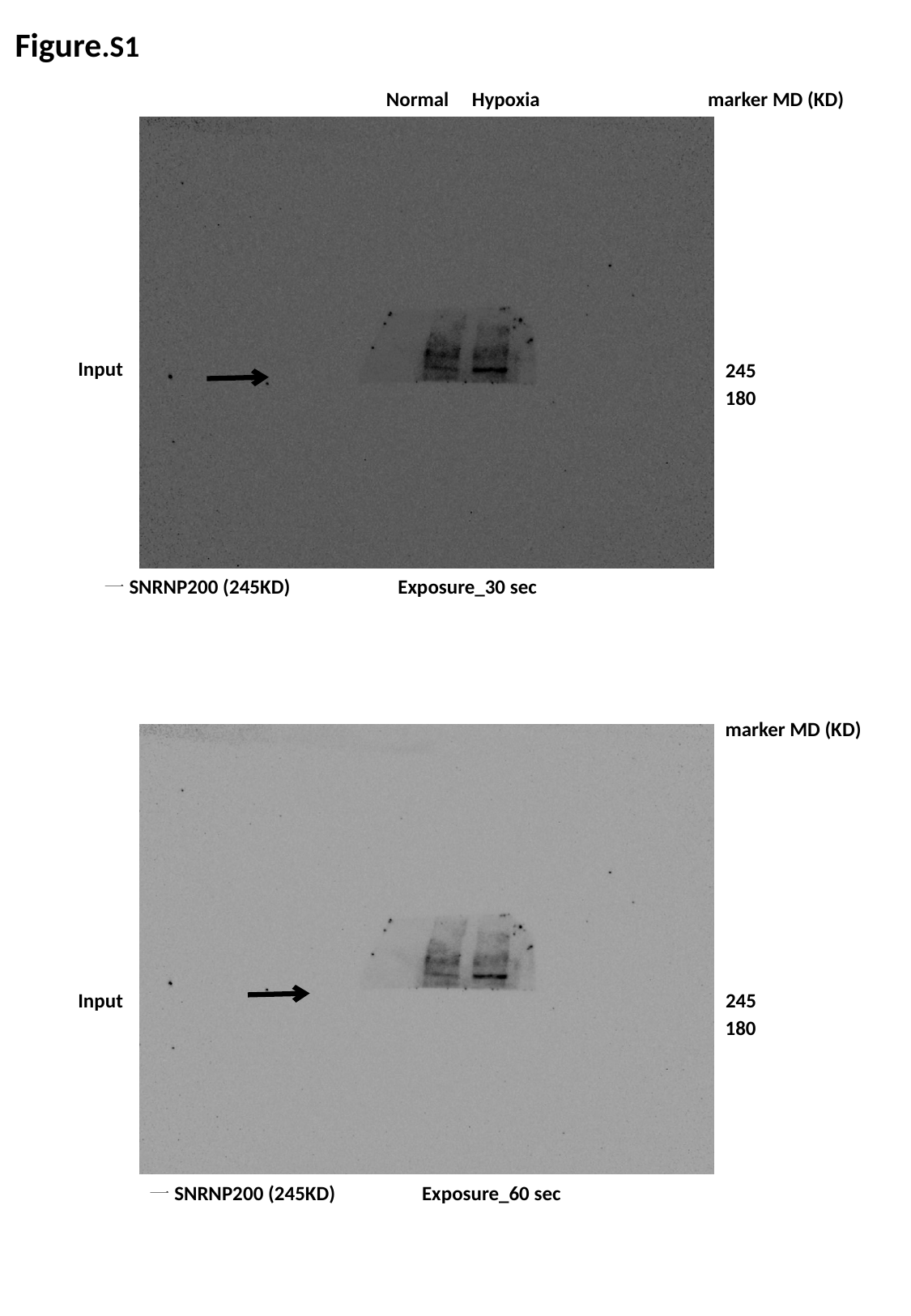

Figure.S1
Normal
Hypoxia
marker MD (KD)
Input
245
180
Exposure_30 sec
一抗：SNRNP200 (245KD)
marker MD (KD)
Input
245
180
一抗：SNRNP200 (245KD)
Exposure_60 sec

## Slide 2
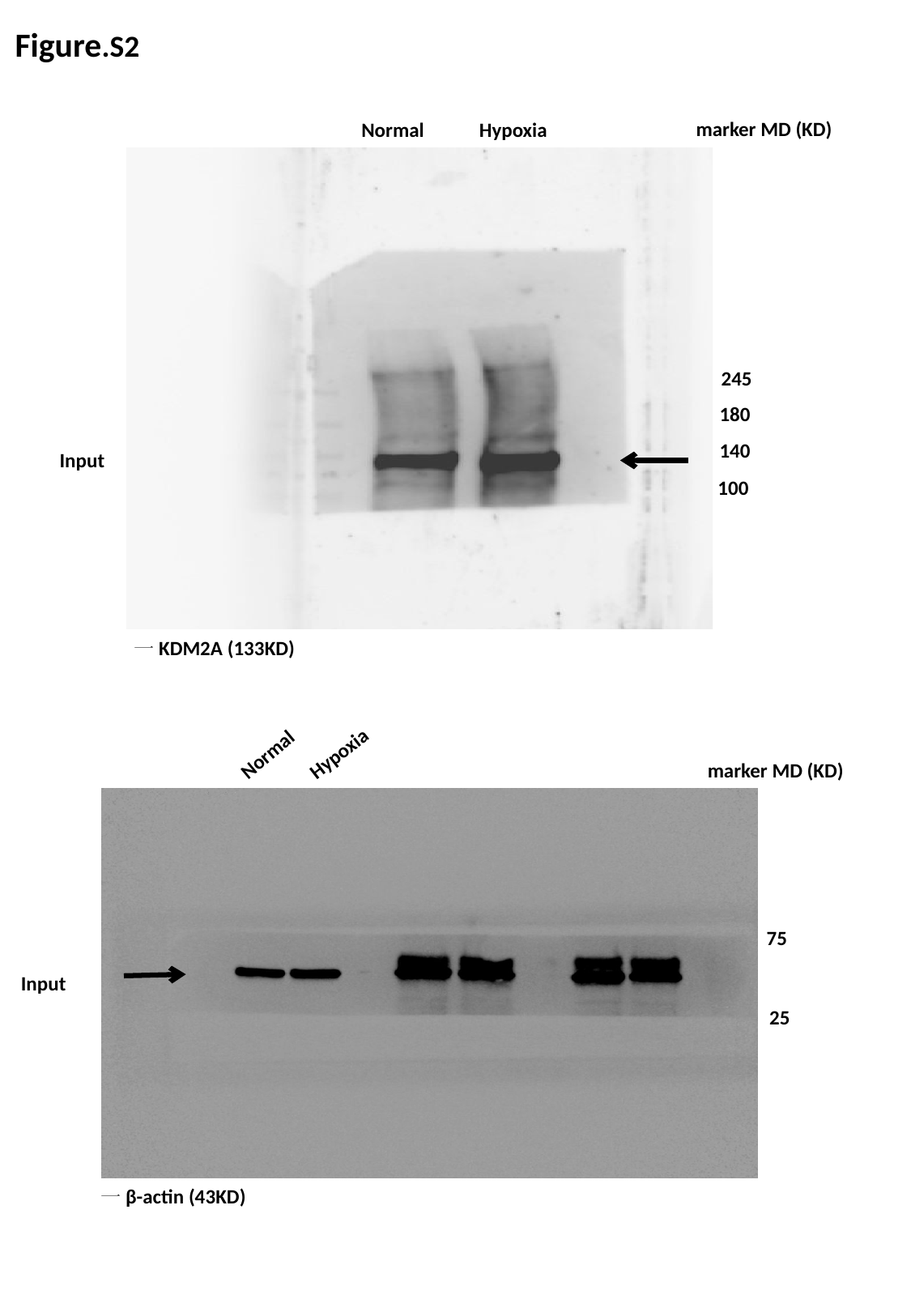

Figure.S2
marker MD (KD)
Normal
Hypoxia
245
180
140
Input
100
一抗：KDM2A (133KD)
Hypoxia
Normal
marker MD (KD)
75
Input
25
一抗：β-actin (43KD)

## Slide 3
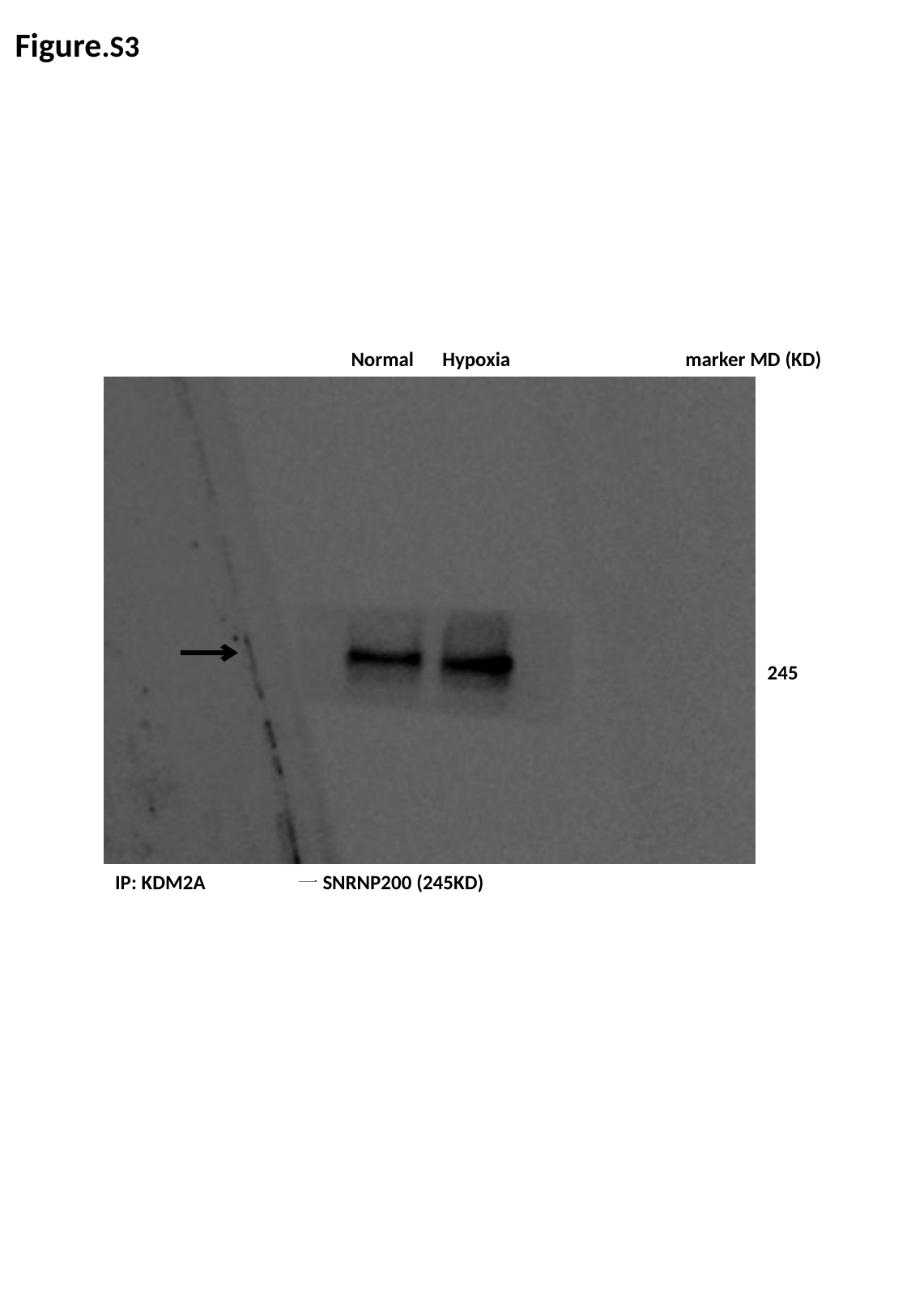

Figure.S3
Normal
Hypoxia
marker MD (KD)
245
IP: KDM2A
一抗：SNRNP200 (245KD)

## Slide 4
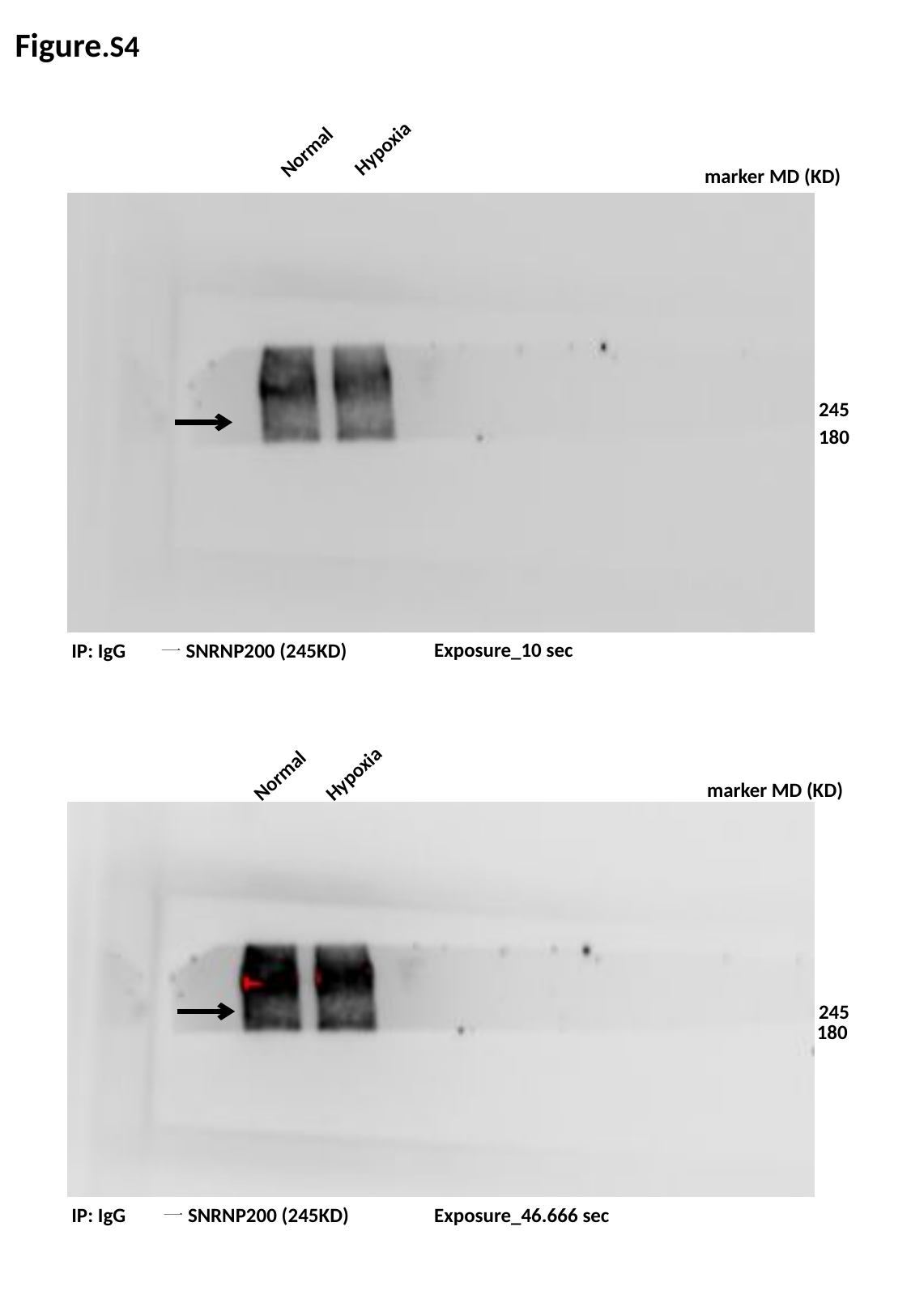

Figure.S4
Hypoxia
Normal
marker MD (KD)
245
180
Exposure_10 sec
IP: IgG
一抗：SNRNP200 (245KD)
Hypoxia
Normal
marker MD (KD)
245
180
IP: IgG
一抗：SNRNP200 (245KD)
Exposure_46.666 sec

## Slide 5
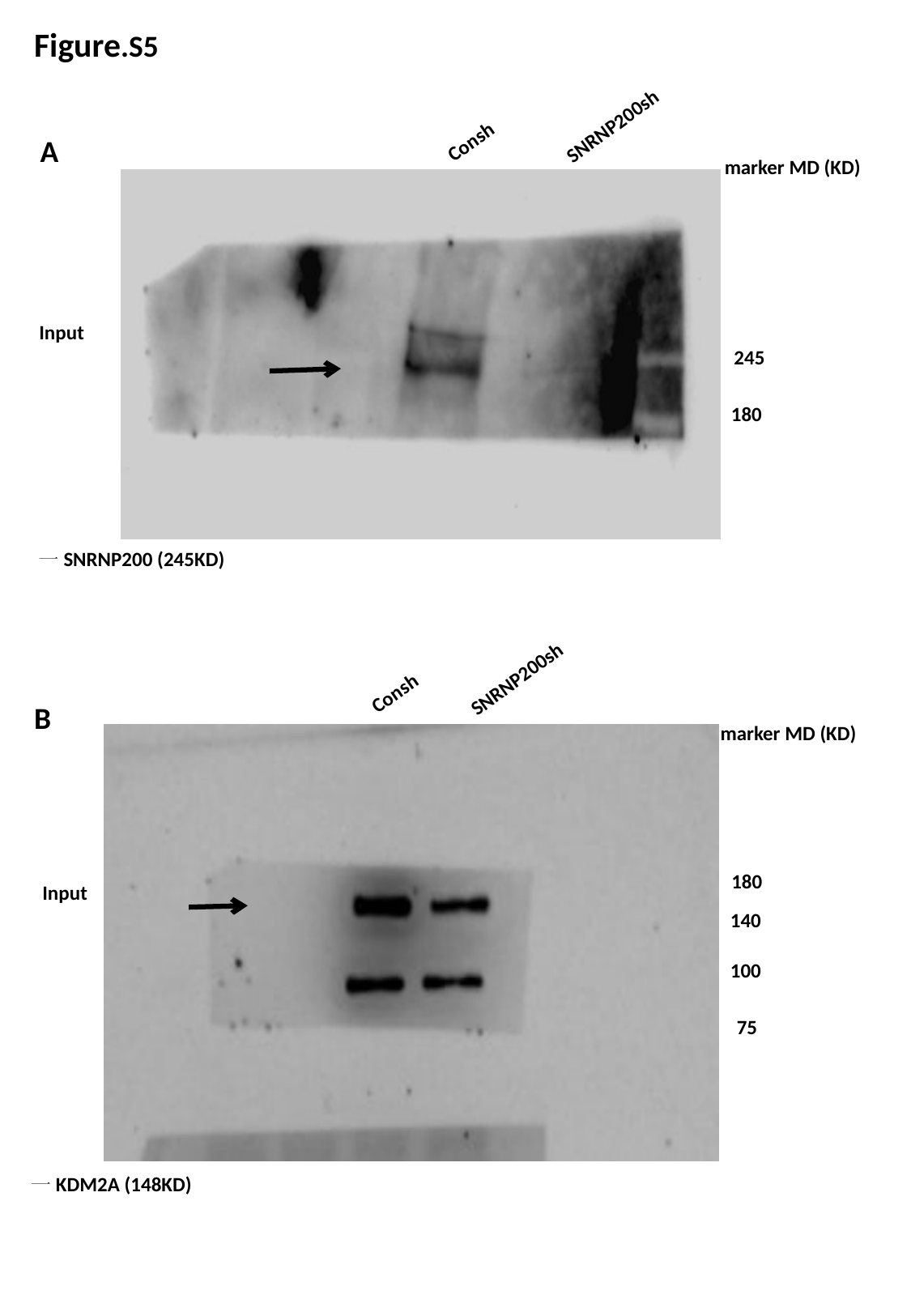

Figure.S5
SNRNP200sh
Consh
marker MD (KD)
Input
245
180
一抗：SNRNP200 (245KD)
A
SNRNP200sh
Consh
B
marker MD (KD)
180
Input
140
100
75
一抗：KDM2A (148KD)

## Slide 6
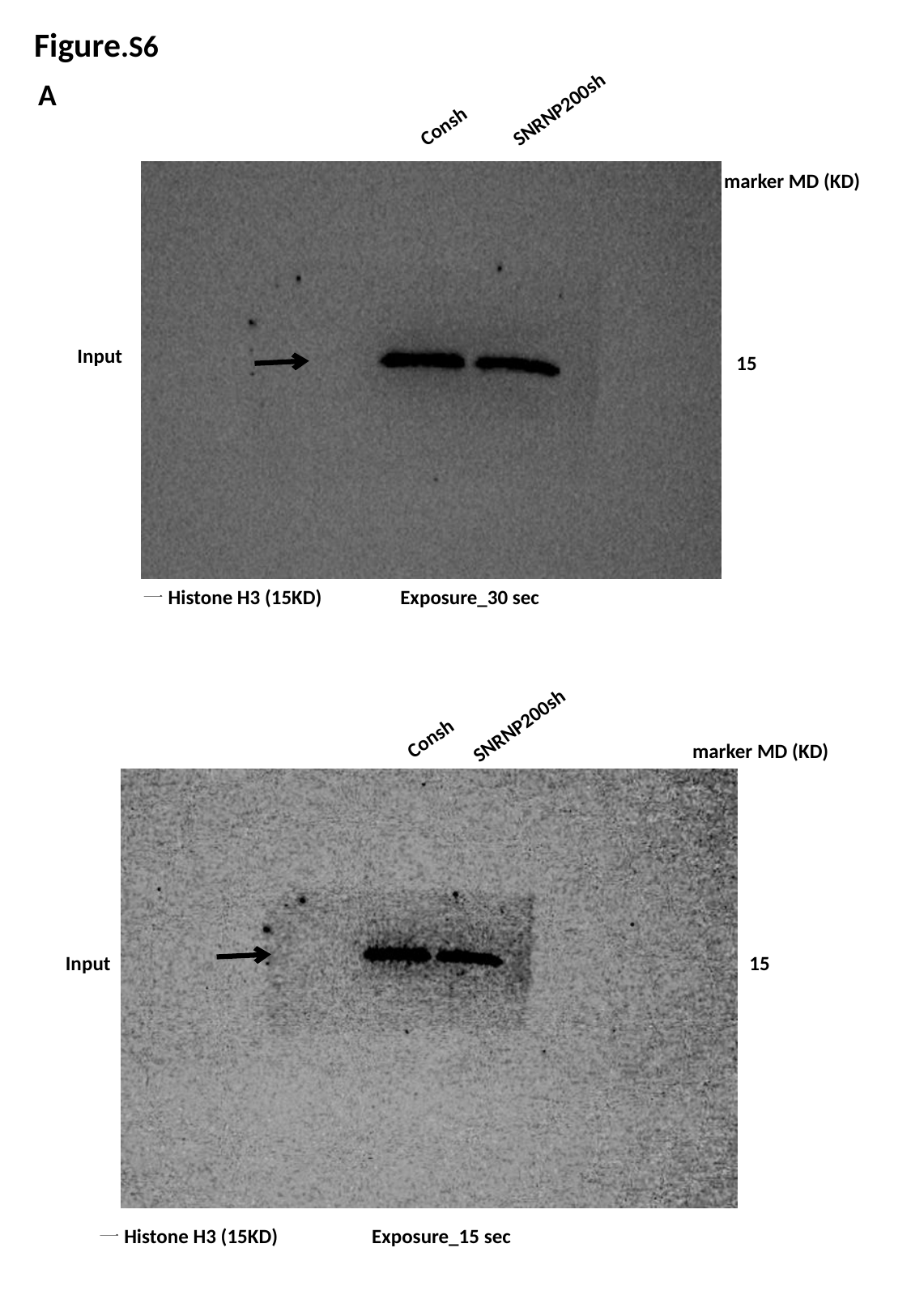

Figure.S6
A
SNRNP200sh
Consh
marker MD (KD)
Input
15
一抗：Histone H3 (15KD)
Exposure_30 sec
SNRNP200sh
Consh
marker MD (KD)
Input
15
一抗：Histone H3 (15KD)
Exposure_15 sec

## Slide 7
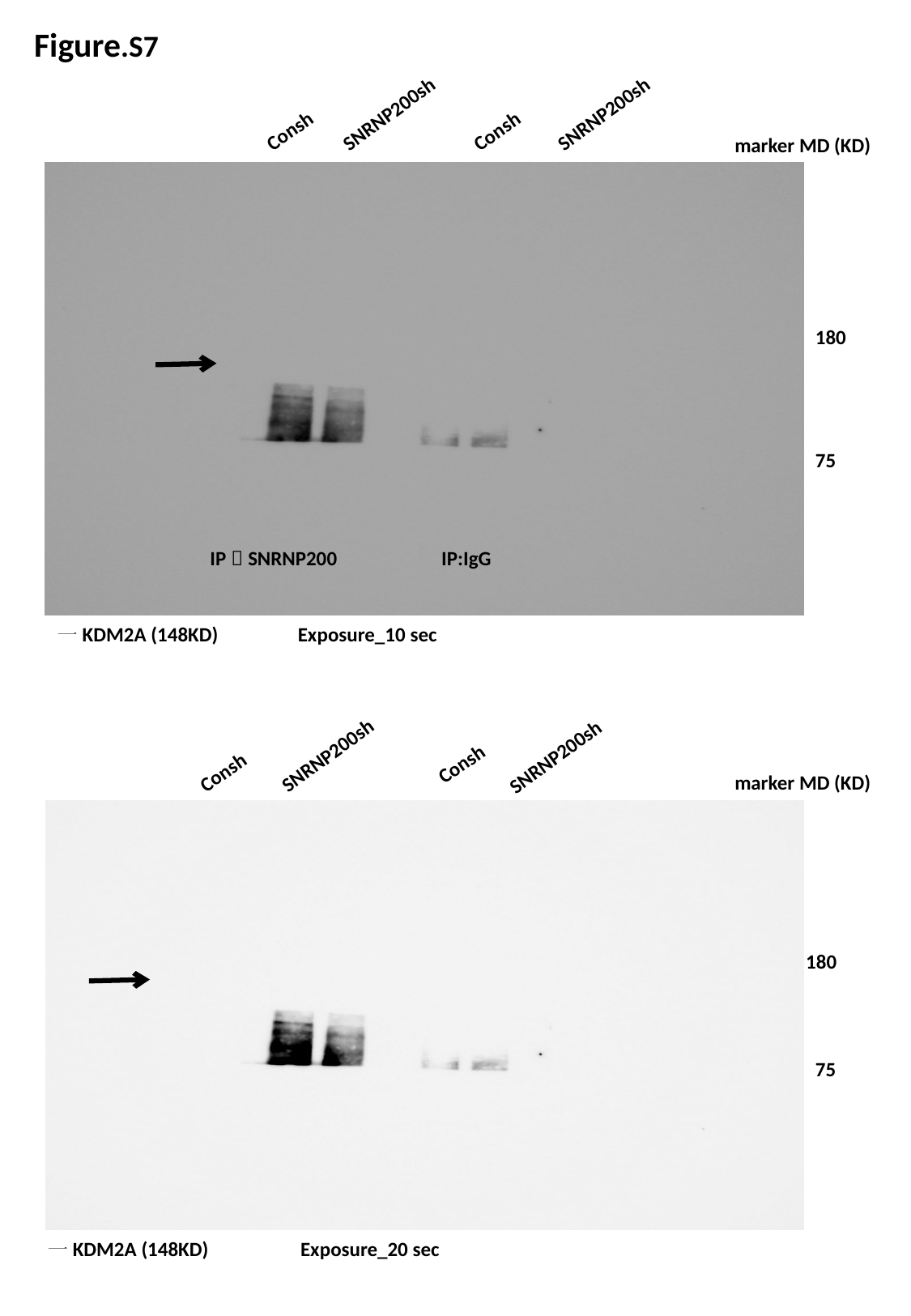

Figure.S7
SNRNP200sh
SNRNP200sh
Consh
Consh
marker MD (KD)
180
75
IP：SNRNP200
IP:IgG
一抗：KDM2A (148KD)
Exposure_10 sec
SNRNP200sh
Consh
SNRNP200sh
Consh
marker MD (KD)
180
75
Exposure_20 sec
一抗：KDM2A (148KD)

## Slide 8
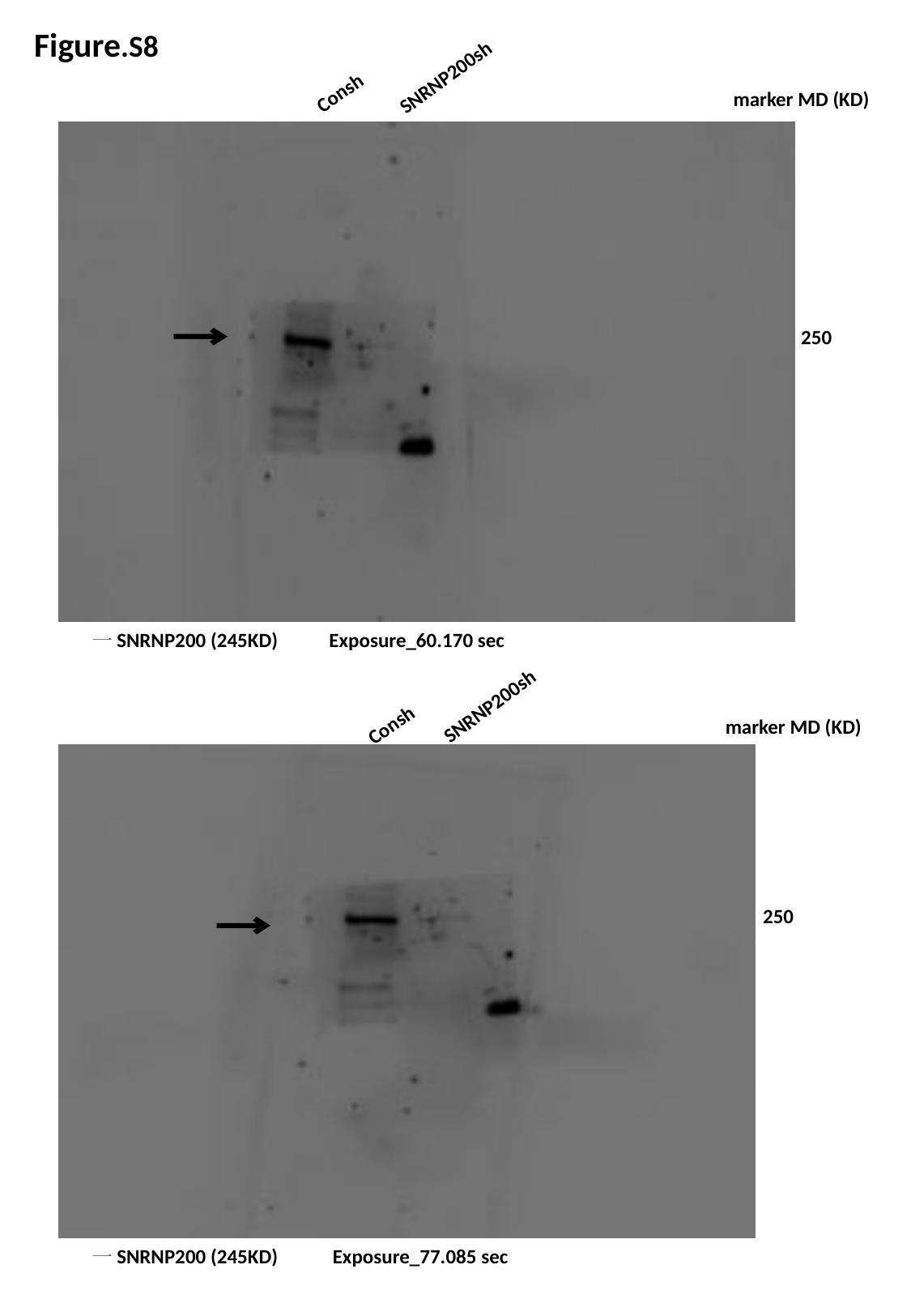

Figure.S8
SNRNP200sh
Consh
marker MD (KD)
250
一抗：SNRNP200 (245KD)
Exposure_60.170 sec
SNRNP200sh
Consh
marker MD (KD)
250
一抗：SNRNP200 (245KD)
Exposure_77.085 sec

## Slide 9
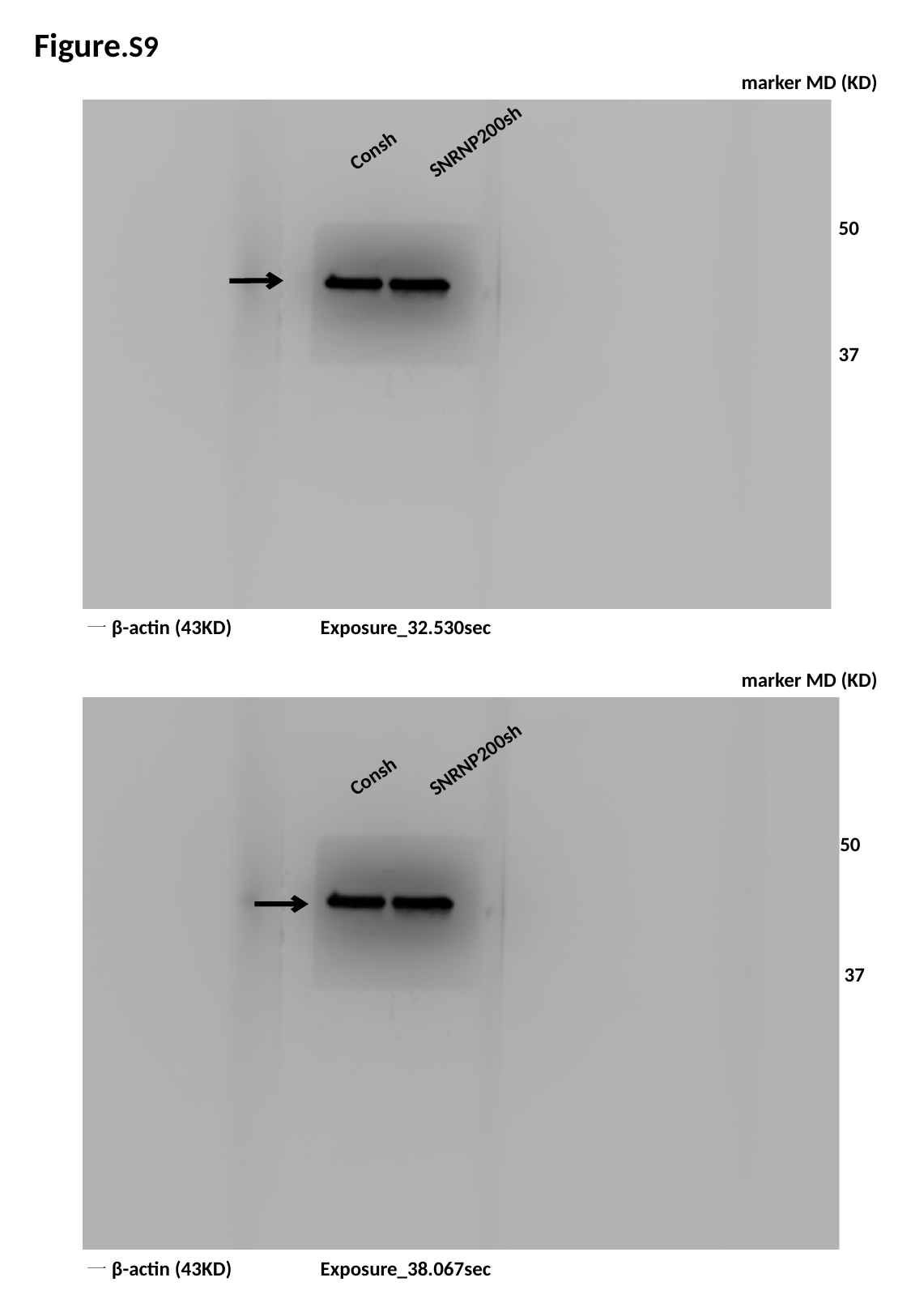

Figure.S9
marker MD (KD)
Consh
SNRNP200sh
50
37
一抗：β-actin (43KD)
Exposure_32.530sec
marker MD (KD)
SNRNP200sh
Consh
50
37
一抗：β-actin (43KD)
Exposure_38.067sec

## Slide 10
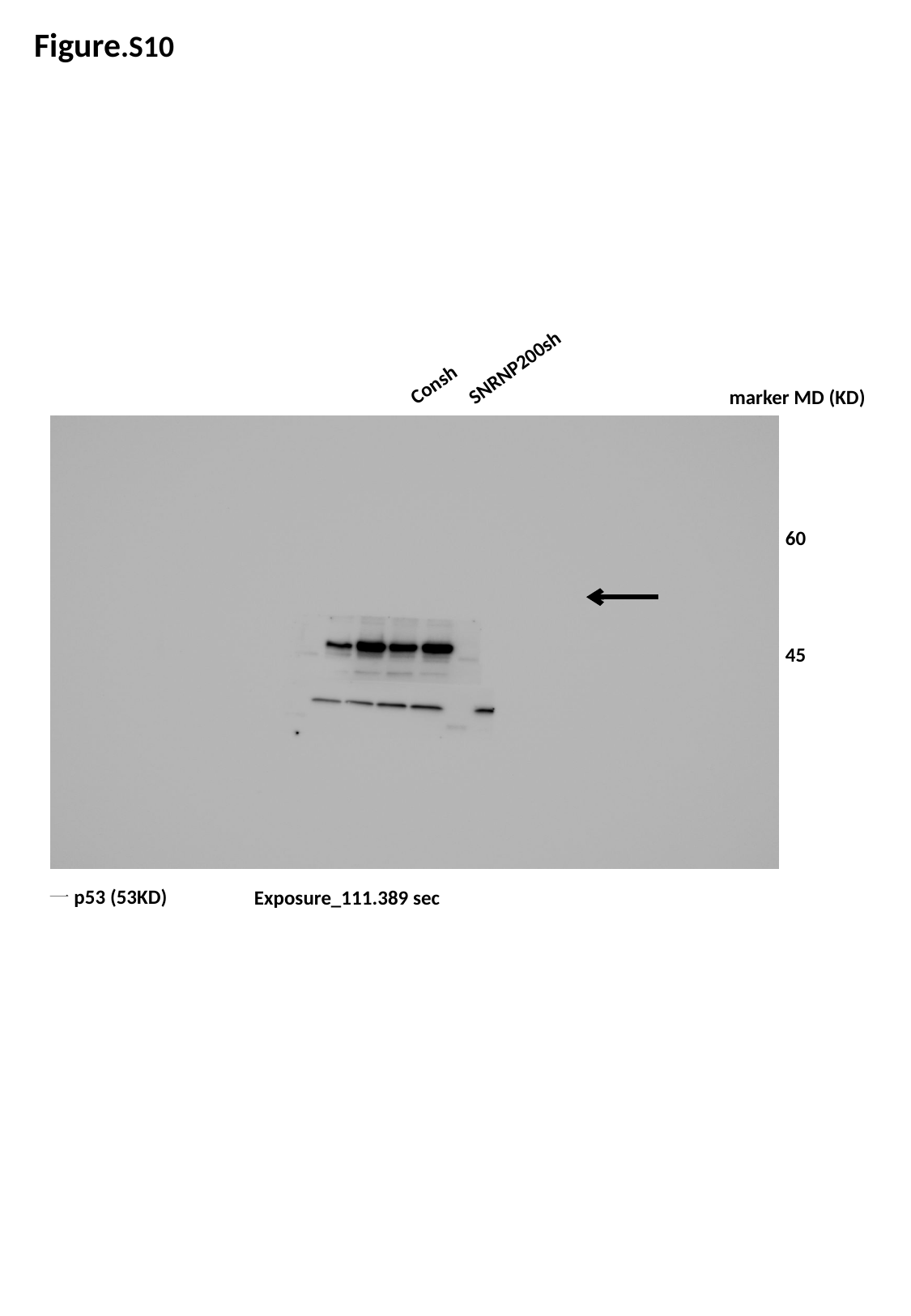

Figure.S10
SNRNP200sh
Consh
marker MD (KD)
60
45
一抗：p53 (53KD)
Exposure_111.389 sec

## Slide 11
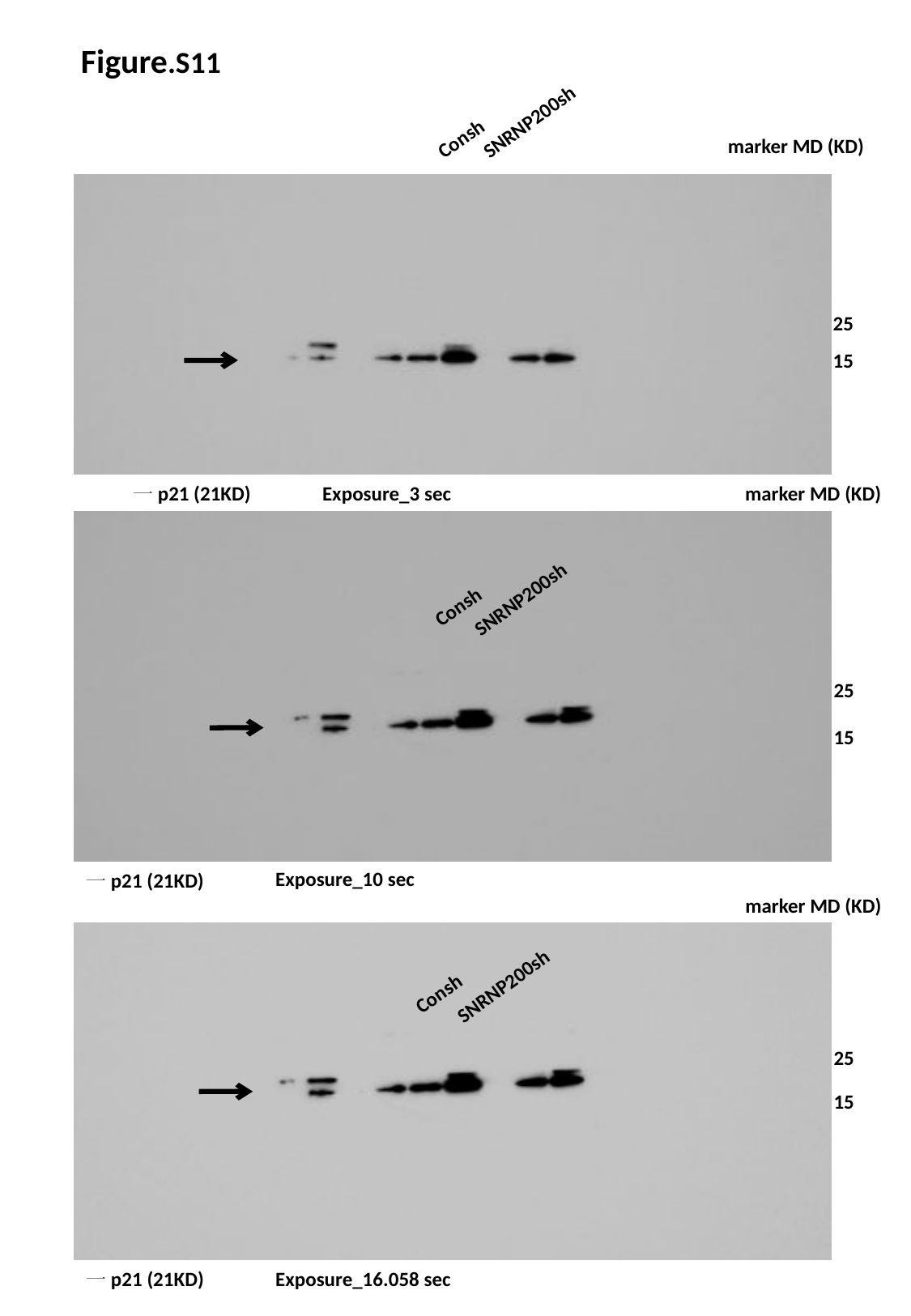

Figure.S11
SNRNP200sh
Consh
marker MD (KD)
25
15
一抗：p21 (21KD)
Exposure_3 sec
marker MD (KD)
Consh
SNRNP200sh
25
15
Exposure_10 sec
一抗：p21 (21KD)
marker MD (KD)
Consh
SNRNP200sh
25
15
一抗：p21 (21KD)
Exposure_16.058 sec

## Slide 12
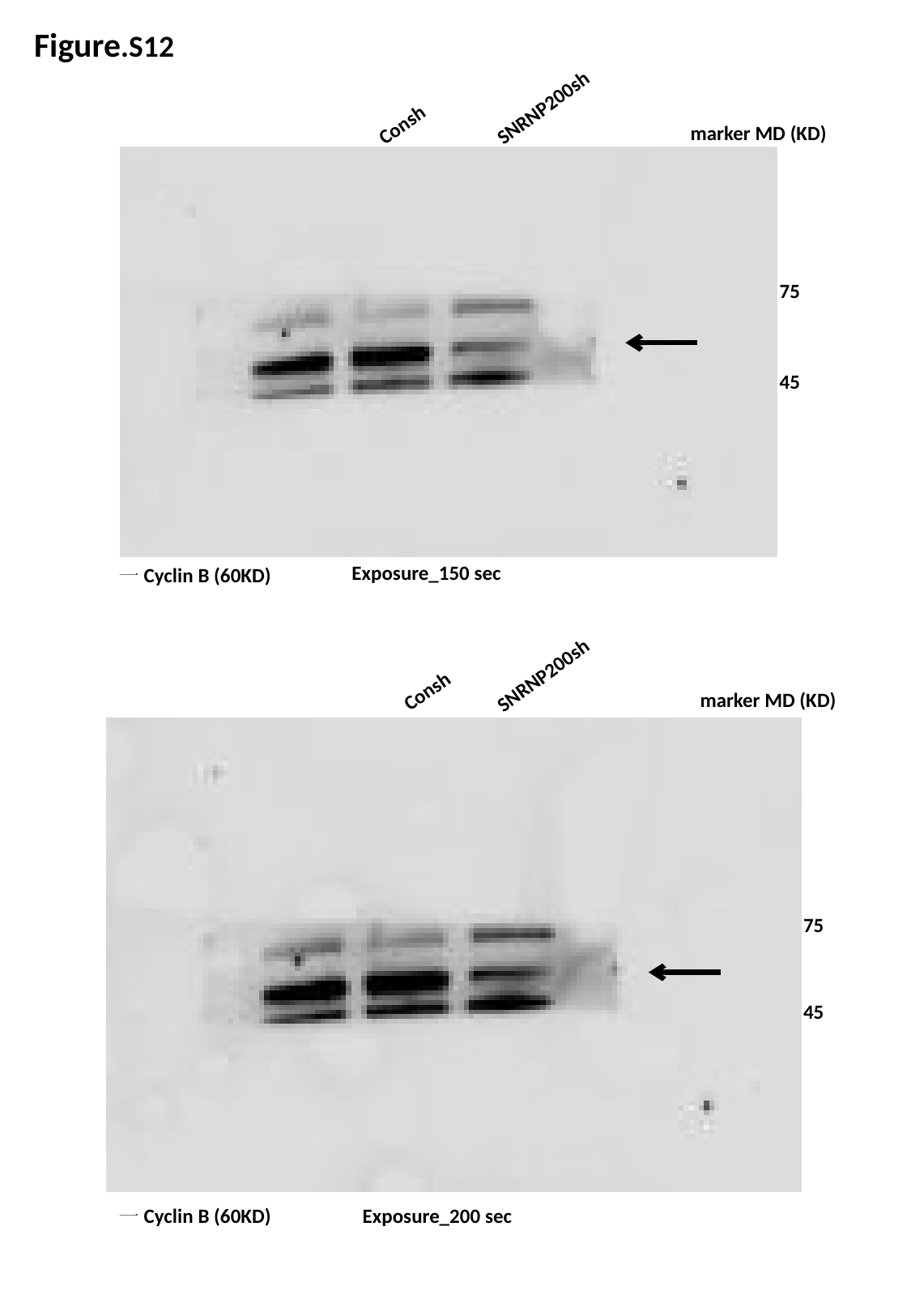

Figure.S12
SNRNP200sh
Consh
marker MD (KD)
75
45
Exposure_150 sec
一抗：Cyclin B (60KD)
SNRNP200sh
Consh
marker MD (KD)
一抗：Cyclin B (60KD)
Exposure_200 sec
75
45

## Slide 13
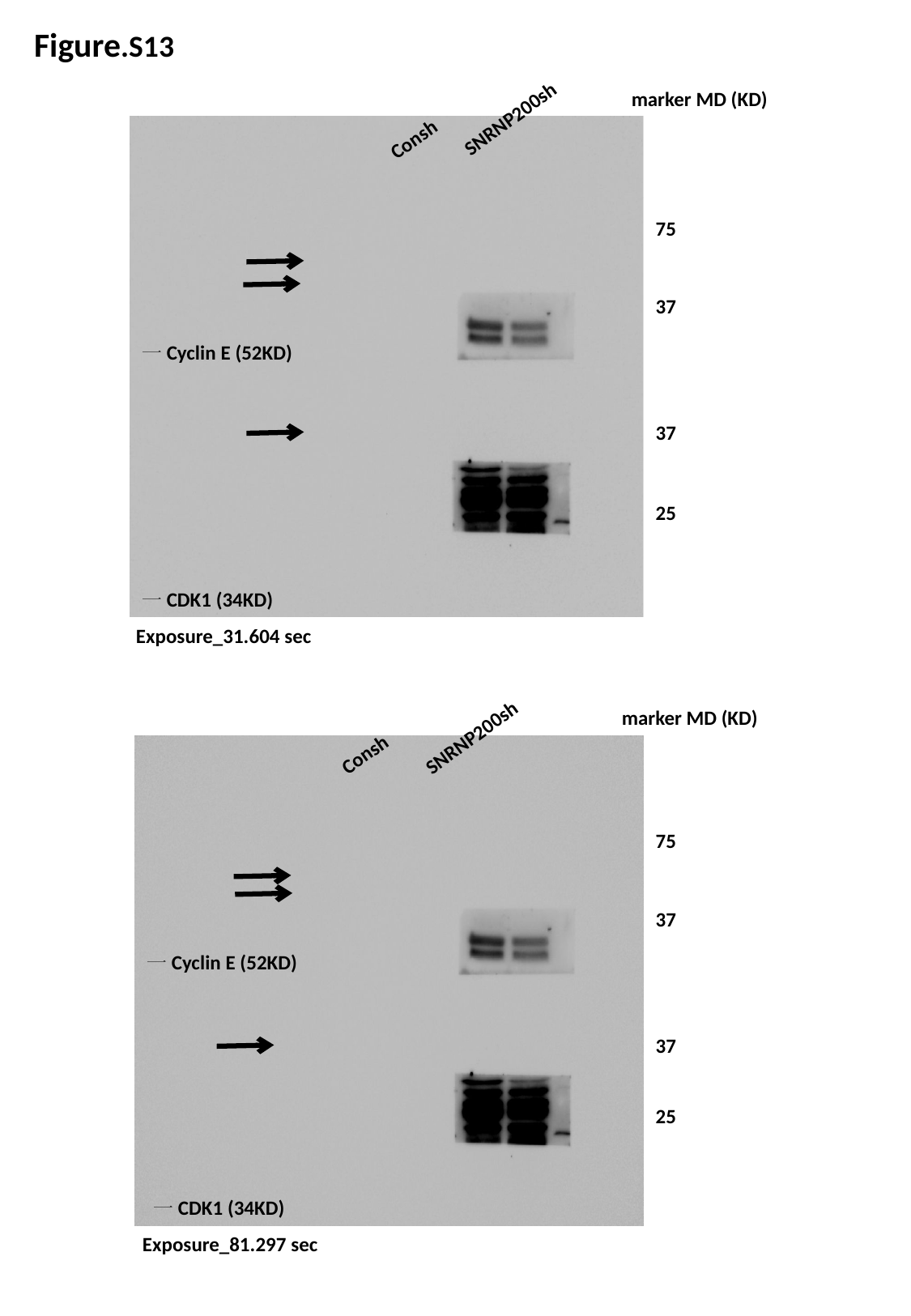

Figure.S13
marker MD (KD)
SNRNP200sh
Consh
75
37
一抗：Cyclin E (52KD)
37
25
一抗：CDK1 (34KD)
Exposure_31.604 sec
marker MD (KD)
SNRNP200sh
Consh
75
37
一抗：Cyclin E (52KD)
37
25
一抗：CDK1 (34KD)
Exposure_81.297 sec

## Slide 14
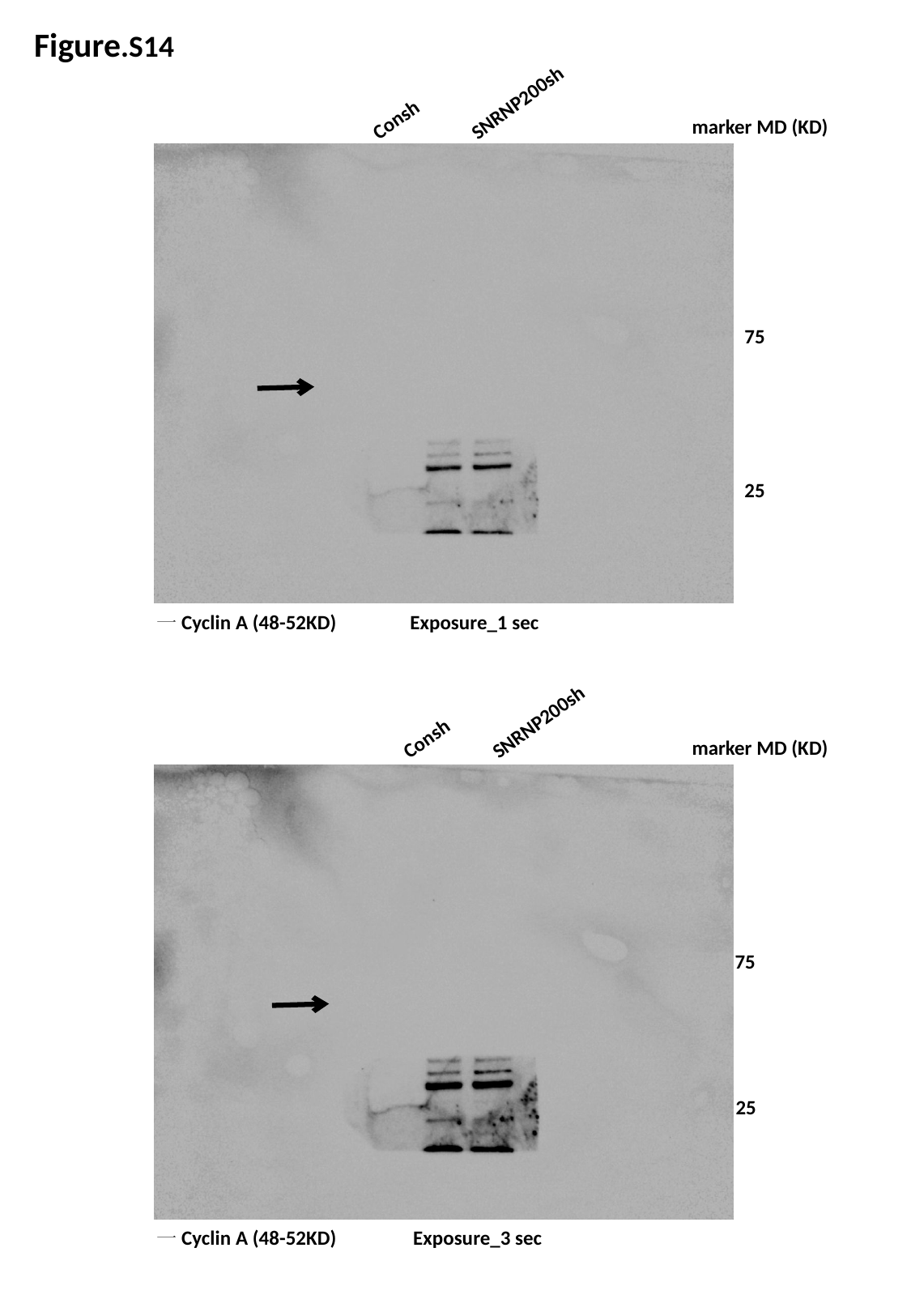

Figure.S14
SNRNP200sh
Consh
marker MD (KD)
75
25
一抗：Cyclin A (48-52KD)
 Exposure_1 sec
SNRNP200sh
Consh
一抗：Cyclin A (48-52KD)
 Exposure_3 sec
marker MD (KD)
75
25

## Slide 15
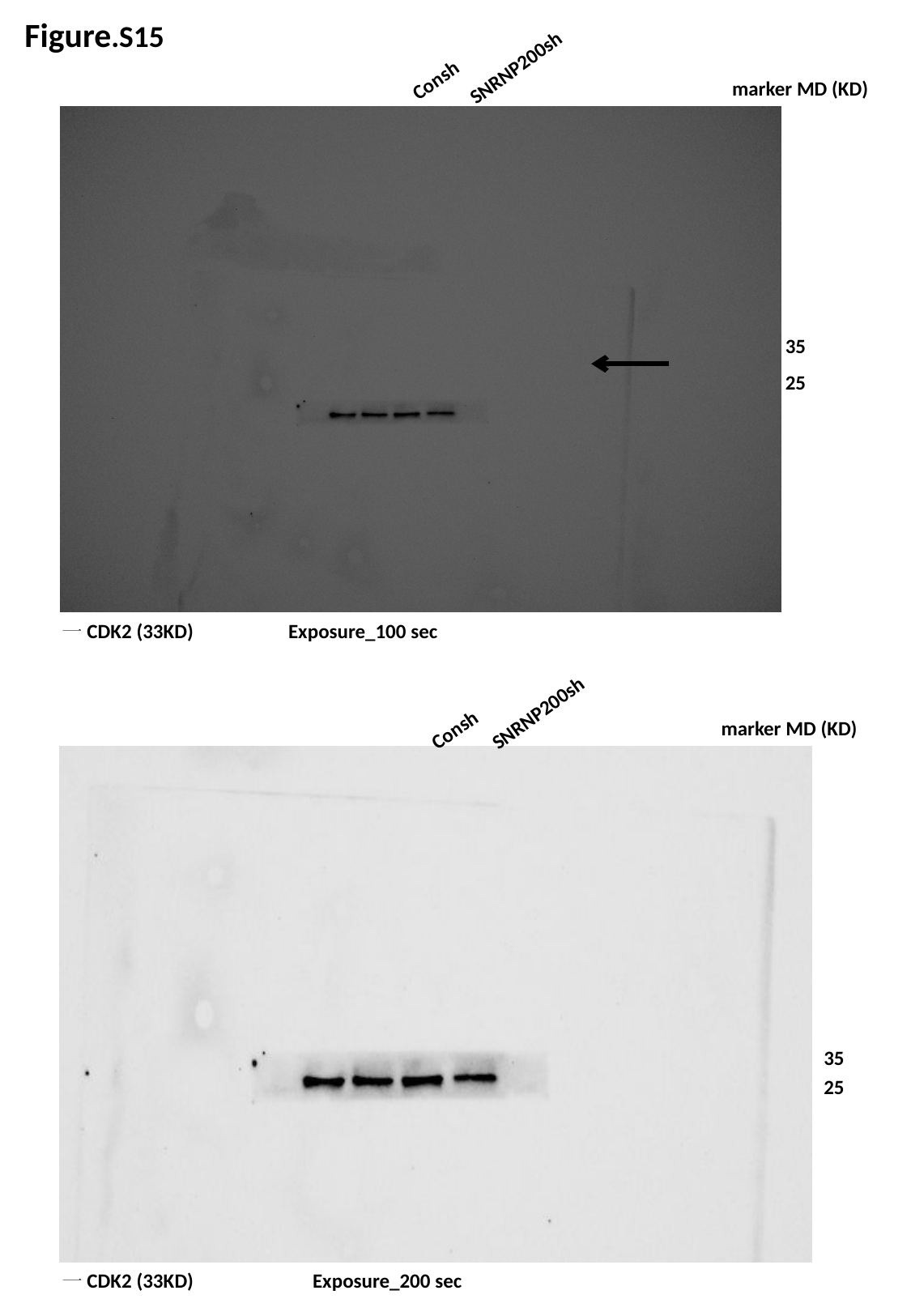

Figure.S15
Consh
SNRNP200sh
marker MD (KD)
35
25
一抗：CDK2 (33KD)
 Exposure_100 sec
SNRNP200sh
Consh
marker MD (KD)
一抗：CDK2 (33KD)
35
25
 Exposure_200 sec

## Slide 16
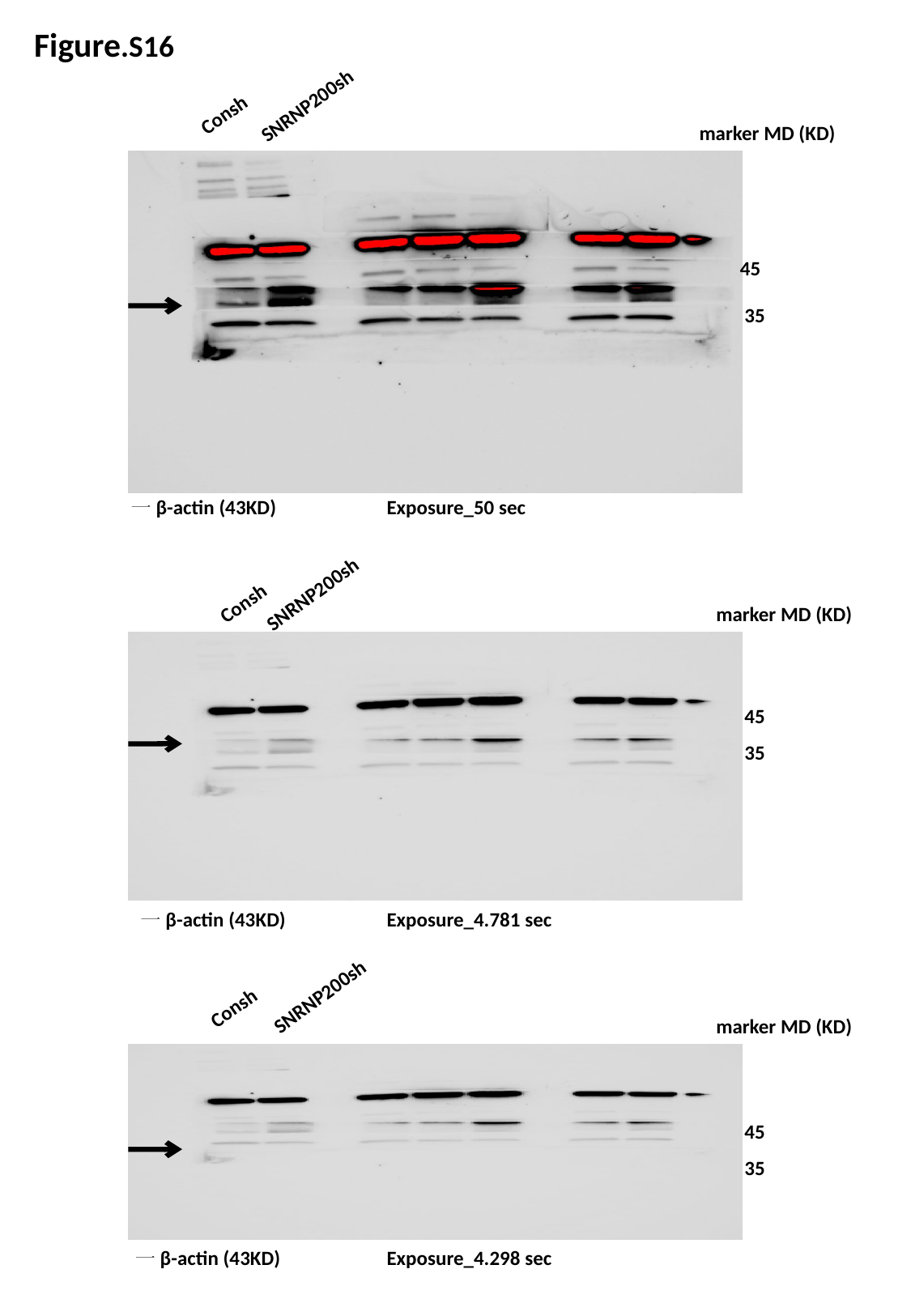

Figure.S16
Consh
SNRNP200sh
marker MD (KD)
45
35
一抗：β-actin (43KD)
 Exposure_50 sec
Consh
SNRNP200sh
marker MD (KD)
45
35
一抗：β-actin (43KD)
 Exposure_4.781 sec
Consh
SNRNP200sh
marker MD (KD)
45
35
一抗：β-actin (43KD)
 Exposure_4.298 sec
